# Two new species of Tardigrada from moss cushions (*Grimmia* sp.) in a xerothermic habitat in northeast Tennessee (USA, North America), with the first identification of males in the genus *Viridiscus*

**DOI:** 10.7717/peerj.10251

**Published:** 2020-11-23

**Authors:** Diane R. Nelson, Rebecca Adkins Fletcher, Roberto Guidetti, Milena Roszkowska, Daria Grobys, Łukasz Kaczmarek

**Affiliations:** 1 Department of Biological Sciences, East Tennessee State University, Johnson City, TN, United States of America; 2 Department of Appalachian Studies, East Tennessee State University, Johnson City, TN, United States of America; 3 Department of Life Sciences, University of Modena and Reggio Emilia, Modena, Italy; 4 Department of Animal Taxonomy and Ecology, Department of Bioenergetics, Adam Mickiewicz University, Poznań, Poland; 5 Department of Bioenergetics, Adam Mickiewicz University, Poznań, Poland; 6 Department of Animal Taxonomy and Ecology, Adam Mickiewicz University, Poznań, Poland

**Keywords:** Dioecious, Eutardigrada, Heterotardigrada, Systematics, Taxonomy, Water bears, Morphology, Molecular technique, Parthenogenesis, Anhydrobiosis

## Abstract

**Background:**

The phylum Tardigrada consists of over 1,300 species that inhabit terrestrial, freshwater and marine environments throughout the world. In terrestrial habitats they live primarily in mosses, lichens, leaf litter and soil, whereas tardigrades in freshwater and marine environments are mainly found in sediments and on aquatic plants. More than 65 species have been previously reported in the state of Tennessee, USA.

**Methods:**

Tardigrades present in moss cushions (*Grimmia* sp.) collected from a xerothermic habitat on the East Tennessee State University campus, Johnson City, TN, USA, were extracted, mounted on slides, identified, and counted. Additional samples of fresh dried moss were used for integrative analyses, including morphological analysis with phase contrast (PCM) and scanning electron microscopy (SEM), as well as molecular analyses of COI, 18S rRNA, 28S rRNA, and ITS-2 of the *Macrobiotus* and *Milnesium* species.

**Results:**

Five species were found, including two species new to science: *Viridiscus miraviridis* sp. nov. and *Macrobiotus basiatus* sp. nov. *Viridiscus miraviridis* sp. nov. differs from other members of the genus mainly by having a different type of dorsal cuticle and some other, more subtle, morphometric characters. In addition to the two new species, *Viridiscus perviridis* and *Viridiscus viridissimus* were present, and males of *Vir. viridissimus* were found for the first time, the first record of males in the genus *Viridiscus*. *Macrobiotus basiatus* sp. nov. is most similar to *Macrobiotus nelsonae*, but it differs from *Mac. nelsonae* mainly by the stylet supports being situated in a more anterior position, shorter and narrower egg processes, and a smaller number of areoles around the egg processes. Moreover, the identification of *Milnesium inceptum* was confirmed as the first record for the USA by analysis of COI.

## Introduction

The phylum Tardigrada consists of over 1,300 species (Guidetti & Bertolani, 2005; Degma & Guidetti, 2007; Degma, Bertolani & Guidetti, 2009–2020) that inhabit terrestrial, freshwater and marine environments throughout the world. In terrestrial habitats they live primarily in mosses, lichens, leaf litter and soil, whereas in freshwater and marine environments tardigrades are found mainly in sediments and on aquatic plants (Nelson, Guidetti & Rebecchi, 2015).

Studies on the distribution of terrestrial and aquatic tardigrades have been conducted primarily in Europe and North America (see e.g., McInnes, 1994; Meyer, 2013; Kaczmarek, Michalczyk & McInnes, 2014; Kaczmarek, Michalczyk & McInnes, 2015; Kaczmarek, Michalczyk & McInnes, 2016; McInnes, Michalczyk & Kaczmarek, 2017). In addition to a few specimens reported by Riggin (1962) and Maucci (1987), surveys of the distribution of tardigrades in Tennessee have been conducted by Nelson (1975) and her students and colleagues (Kathman & Nelson, 1987; Nelson, Kincer & Williams, 1987; Nelson & McGlothlin, 1993; Nelson & McGlothlin, 1996; Kendall-Fite & Nelson, 1996; Guidetti, 1998; Bertolani, Marley & Nelson, 1999; Guidetti, Bertolani & Nelson, 1999; Nelson, Marley & Bertolani, 1999; Nelson & Adkins, 2001; Bartels & Nelson, 2006; Bartels & Nelson, 2007; Bartels & Nelson, 2012; Bartels et al., 2007; Bartels et al., 2008; Bartels et al., 2009; Bartels et al., 2011; Bartels, Nelson & Exline, 2011; Bartels et al., 2014; Bartels, Mormino & Nelson, 2017; Nelson & Bartels, 2007; Nelson & Bartels, 2013; Nelson, Bartels & Guil, 2019). Thus far for the state of Tennessee, more than 65 species have been reported (Kaczmarek, Michalczyk & McInnes, 2016; Meyer, 2013).

The genus *Viridiscus* was recently established by Gąsiorek & Michalczyk in Gąsiorek et al. (2019) in an integrative analysis of the “*arctomys* group” of *Echiniscus*, in which five new genera were erected. The widely distributed “*Echiniscus viridis* group” (now *Viridiscus*), with the very characteristic fully or partially green cuticle and lacking all lateral and dorsal appendages except cirrus *A* currently includes six species: *Viridiscus clavispinosus* (Fontoura, Pilato & Lisi, 2011), *Viridiscus perviridis* (Ramazzotti, 1959), *Viridiscus rufoviridis* (Du Bois Reymond Marcus, 1944), *Viridiscus viridianus* (Pilato, Fontoura & Lisi, 2007), *Viridiscus viridis* (Murray, 1910), and *Viridiscus viridissimus* (Péterfi, 1956). In this paper, we provide a description of a new species of *Viridiscus*, and the first confirmation of the presence of males in the genus.

We also provide the first record of *Milnesium inceptum* Morek et al., 2019 from the USA (Tennessee, Washington County), confirmed by molecular analysis. *Milnesium* Doyère, 1840 was originally believed to be a monospecific genus, but multiple species have been described in recent years with over 40 known species to date (Morek et al., 2020).

In addition, based on an integrative approach, we also provide a description of a new species in the genus *Macrobiotus*. The cosmopolitan genus *Macrobiotus* C.A.S. Schultze, 1834, the first described genus of tardigrades, comprises a large complex of species, some of which have been allocated to several new genera in recent years.

## Materials & Methods

### Sample processing

**Figure 1:**
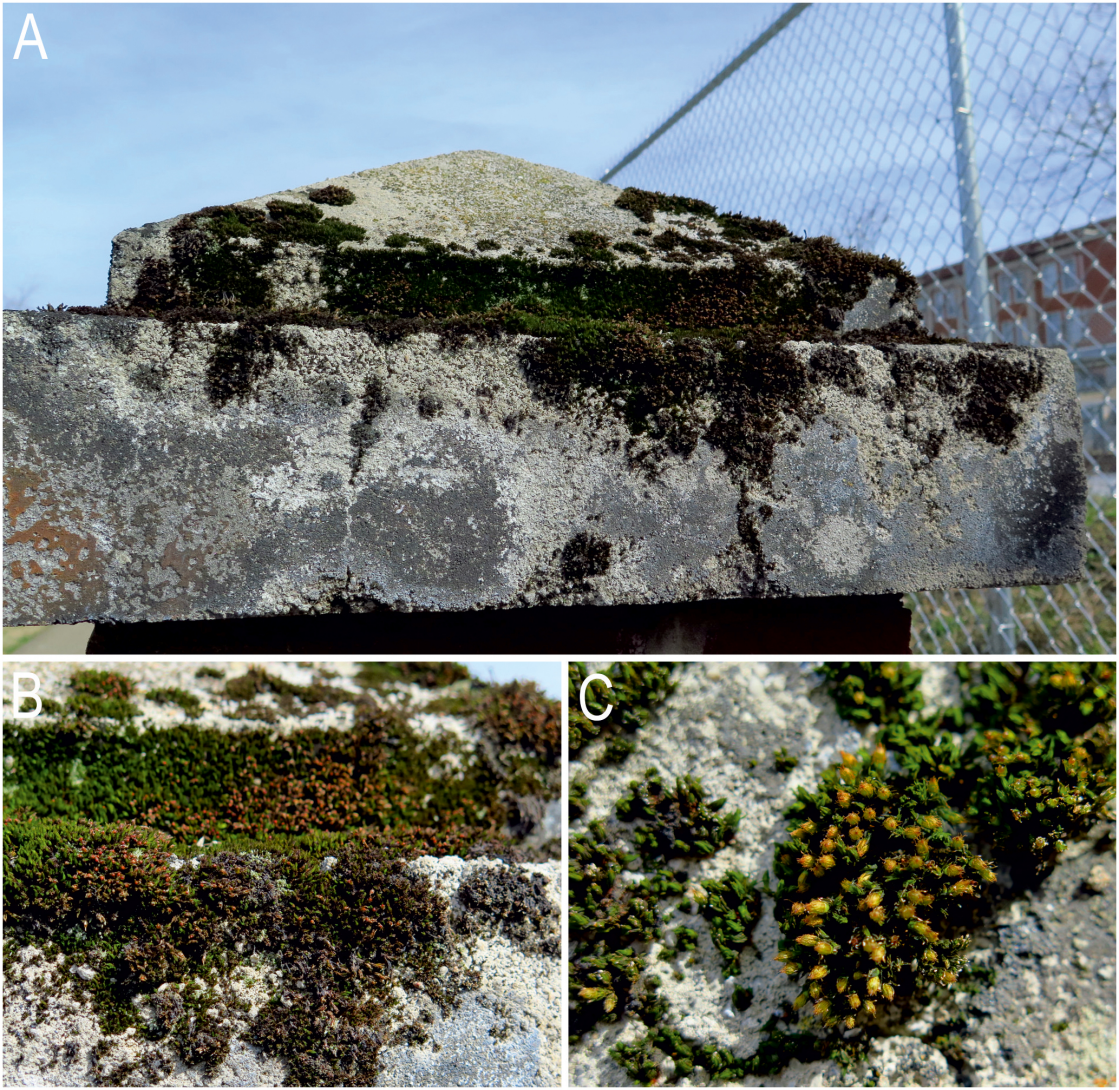
Sampling site and type locality of *Viridiscus miraviridis* sp. nov. and *Macrobiotus basiatus* sp. nov. in moss cushions (*Grimmia* sp.) on the campus of East Tennessee State University, Johnson City, Tennessee. (A) Overall view of one of the brick fence posts on the campus. (B) Close up of the concrete cap on the fence post. (C) Close up of the moss showing the cushion growth form and leaf structures. Photo credit: Diane R. Nelson.

In a previous study (Nelson & Adkins, 2001), 60 samples of the moss *Grimmia* sp. were collected from vertical surfaces of concrete caps on six brick fence posts (10 samples per post), fully exposed to sun, wind and precipitation, located on the north side of the East Tennessee State University campus (Johnson City, Washington County, Tennessee, USA) (Figs. 1A–1C), from 14 May 1995 to 30 September 1995 (for more details see Nelson & Adkins, 2001).

The samples were soaked in tap water in a small jar for 30 min, then agitated vigorously. Afterwards, the sample was poured into a beaker through a wire screen to remove the moss. When the beaker contents settled, the top layer of water was decanted, and boiling alcohol was added to preserve the sample. The beaker contents were then poured through a 45 µm sieve. The remaining sample on the sieve was washed into a petri dish and examined for tardigrades under a stereomicroscope. All tardigrades and eggs found in each sample were extracted with an Irwin loop. To describe the two new species with an integrative approach, additional samples of freshly dried moss from the same posts were collected in 2019 and mailed to Adam Mickiewicz University, Poznań, Poland, for processing, imaging and morphometrics.

### Microscopy and imaging

Specimens from the previous study were mounted on microscope slides in Hoyer’s mounting medium, with the coverslip sealed with epoxy paint when dry. All specimens and eggs were identified to species level with phase contrast light microscopy (PCM). Microphotographs of specimens from the fresh samples were obtained with Olympus BX41 associated with an ARTCAM–300Mi digital camera (Olympus Corporation, Shinjuku–ku, Japan). A total of 10 adults of *Viridiscus miraviridis* sp. nov. and 25 specimens and 15 eggs of *Macrobiotus basiatus* sp. nov. from the fresh samples were prepared for SEM imaging according to Roszkowska et al. (2018). These animals and eggs were examined under high vacuum in a Hitachi S3000N Scanning Electron Microscope.

All figures were assembled in *Corel Photo-Paint 17*. For deep structures that could not be fully focused in a single photograph, a series of 2–10 images were taken every ca. 0.5 µm and then assembled into a single stacked image manually in *Corel Photo-Paint 17*.

### Morphometrics and morphological nomenclature

Structures were measured only if their orientation was suitable. Body length was measured from the anterior extremity to the end of the body, excluding the hind legs. The types of buccal-pharyngeal apparatuses and claws were classified according to Pilato & Binda (2010). The terminology used to describe the genus *Viridiscus* followed Gąsiorek et al. (2019). Lengths of the claw branches for *Viridiscus* were measured from the base of the claw to the top of the branch. The *sp* index, the ratio of the length of a given structure to the length of the scapular plate (scp), was calculated according to Dastych (1999). All measurements of adults and eggs of *Macrobiotus* were prepared according to Kaczmarek & Michalczyk (2017). Terminology describing the oral cavity armature followed Michalczyk & Kaczmarek (2003). The macroplacoid length sequence was determined according to Kaczmarek et al. (2014). The *pt* ratio, the length of a given structure to the length of the buccal tube expressed as a percentage (Pilato, 1981), was also calculated. The age structure of the *Viridiscus* populations was determined according to Bertolani et al. (1984): 1st instar, anus and gonopore absent, 2 claws per leg present; 2nd instar, anus present but gonopore absent or rudimentary, 4 claws per leg present; adult, anus and gonopore present, 4 claws per leg present.

Morphometric data were handled using the “Parachela” ver. 1.1 and “Echiniscoidea” ver. 1.1 templates available from the Tardigrada Register (Michalczyk & Kaczmarek, 2013). Tardigrade taxonomy followed Bertolani et al. (2014). Genera were abbreviated according to Perry, Miller & Kaczmarek (2019).

### Genotyping

All specimens of *Macrobiotus* and *Milnesium* were preliminarily identified using light microscopy before DNA extraction. DNA was extracted from individual animals following a *Chelex*^®^*100* resin (*Bio-Rad*) extraction method (Casquet, Thebaud & RG, 2012) modified to obtain tardigrade exoskeletons according to Kaczmarek et al. (2019). For *Macrobiotus* specimens, we sequenced four DNA fragments differing in mutation rates: the cytochrome oxidase subunit I (COI, mtDNA), the internal transcribed spacer (ITS-2, nDNA), the large ribosome subunit (28S rRNA, nDNA) and the small ribosome subunit (18S rRNA, nDNA). For *Milnesium* specimens we sequenced only the COI fragment. All fragments were amplified and sequenced with the protocols described in Kaczmarek et al. (2019). Amplification of 28S rRNA and 18S rRNA fragments was conducted in the reaction mixture with addition of 3% DMSO. Primers used for PCR, and later sequencing, with their original references, are listed in Table 1. PCR programs used for the amplification of COI, ITS-2, 28S rRNA and 18S rRNA are listed in Table 2. For analysis of COI, PCR products were purified by thermosensitive Exonuclease I and FastAP Alkaline Phosphatase (Fermentas, Thermo Scientific), whereas ITS-2, 28S rRNA and 18S rRNA (after excision from the gel) were treated with NucleoSpin Gel and PCR Clean-up Kit (MACHERY-NAGEL). For sequencing COI, ITS-2 and 28S rRNA, the same primers were used as in the amplification reaction, while a few additional primers were required to sequence the 18S rRNA (see Table 1).

**Table 1:** Primers used for amplification and sequencing of DNA fragments.

**DNA fragment**	**Application**	**Direction**	**Code**	**Sequence (5′–3′)**	**Reference**
COI	PCR & sequencing	Forward	LCO 1490	GGTCAACAAATCATAAAGATATTGG	Folmer et al. (1994)
		Reverse	HCO 2198	TAAACTTCAGGGTGACCAAAAAATCA	
ITS-2	PCR & sequencing	Forward	ITS2_Eutar_Ff	CGTAACGTGAATTGCAGGAC	Stec, Kristensen & Michalczyk (2018)
		Reverse	ITS2_Eutar_Rr	TGATATGCTTAAGTTCAGCGG	
28S rRNA	PCR & sequencing	Forward	28SF0001	ACCCVCYNAATTTAAGCATAT	Mironov, Dabert & Dabert (2012)
		Reverse	28SR0990	CCTTGGTCCGTGTTTCAAGAC	
18S rRNA	PCR & sequencing	Forward	Euk-A	AACCTGGTTGATCCTGCCAGT	Koid et al. (2012)
		Reverse	Euk-B	GATCCTTCTGCAGGTTCACCTAC	
	sequencing	Forward	fw390	AATCAGGGTTCGATTCCGGAGA	Dabert et al. (2010)
		Forward	for_770	ACTTTGAAAAAATTAGAGTGC	
		Reverse	rev930	GACGGTCCAAGAATTTCAC	
		Reverse	rev_1460	CATCACAGACCTGTTATTGC	

The sequences were edited and manually checked against non-conservative alignments using BioEdit, version 7.0.5. (Hall, 1999). The 18S rRNA sequence was assembled using the CAP contig assembly program implemented in BioEdit. All sequences were submitted to GenBank (see ‘Results’ section).

We were not able to obtain DNA from the *Viridiscus* specimens since more than one morphologically very similar species of *Viridiscus* were present and insufficient numbers were present in the new samples from the study sites.

### Comparative molecular analysis

The obtained sequences were analysed by Standard Nucleotide BLAST to confirm their uniqueness. Then, a comparison was performed with the COI sequence of the genus *Milnesium*, as well as COI, ITS-2, 18S rRNA, and 28S rRNA sequences of the genus *Macrobiotus*, deposited in GenBank, using only the sequences of good quality and length. All sequences were aligned with the ClustalW Multiple Alignment tool (Thompson, Higgins & Gibson, 1994) implemented in BioEdit. Alignment sequences were trimmed to 759, 678, 276 and 518 bp for 18S rRNA (21 sequences, 17 species), 28S rRNA (18 sequences, 11 species), ITS2 (21 sequences, 12 species) and COI (32 haplotypes, 59 sequences, 16 species) barcodes, respectively, and calculation for the uncorrected p-distances was performed using MEGA X (Kumar et al., 2018). Uncorrected pairwise distances are provided in Table S1. Table S2 provides the name, accession number and link for the third-party database where the sequences have been deposited.

**Table 2:** PCR programs used for the amplification of COI, ITS-2, 28S rRNA and 18S rRNA.

	**COI and ITS-2**			**28S rRNA**			**18S rRNA**		
**Step**	**Cycles**	**Time [min.:sec.]**	**Temp. [°C]**	**Cycles**	**Time [min:sec]**	**Temp. [°C]**	**Cycles**	**Time [min:sec]**	**Temp. [°C]**
initial denaturation	–	03:00	98	–	05:00	98	–	05:00	98
denaturation	40	00:15	98	40	00:30	98	40	00:30	98
annealing		00:30	50		01:00	50		01:00	60
extension		00:30	72		01:00	72		01:30	72
final extension	–	07:00	72	–	07:00	72	–	07:00	72

### Comparative material

For comparison with the new species, the following specimens were used: *Vir. viridis* collected in Hawaii (Schuster collection at the Bohart Museum, University of California-Davis, USA); *Vir. viridianus* (paratype 060511PF) collected in Azores Is. (Fontoura Collection, Department of Zoology and Anthropology, University of Porto, Portugal); *Vir. rufoviridis* collected in Ecuador (slides 13775, 13779, 13814, 13817); *Vir. viridissimus* collected in Johnson City, TN, USA (slides 7731, 7735, 7843); *Vir. viridis* collected in Ecuador (slide 13821); *Vir. perviridis* collected in Johnson City, TN, USA (slide 7744) and in Italy (slide 4564) (Maucci collection at the Natural History Museum of Verona, Italy); and holotype and paratypes of *Macrobiotus nelsonae* (Guidetti, 1998) collected on Roan Mountain, Tennessee (slides 5N05A-S10) (Bertolani Collection, Department of Life Science, University of Modena and Reggio Emilia, Italy).

In addition to the key to *Viridiscus* in Fontoura, Pilato & Lisi (2011), the following original descriptions or redescriptions, as well as more recent papers, were used: Murray (1910), Du Bois Reymond Marcus (1944), Péterfi (1956), Ramazzotti (1959), Pilato, Fontoura & Lisi (2007); Pilato, Fontoura & Lisi (2008), and Morek et al. (2019).

For the differential diagnosis of the new *Macrobiotus* species, the following original descriptions or redescriptions were used: Maucci (1954), Guidetti (1998), Binda et al. (2001) and Meyer et al. (2017).

For the correct identification of *Mil. inceptum*, we used the original description in Morek et al. (2019).

The electronic version of this article in Portable Document Format (PDF) will represent a published work according to the International Commission on Zoological Nomenclature (ICZN), and hence the new names contained in the electronic version are effectively published under that Code from the electronic edition alone. This published work and the nomenclatural acts it contains have been registered in ZooBank, the online registration system for the ICZN. The ZooBank LSIDs (Life Science Identifiers) can be resolved and the associated information viewed through any standard web browser by appending the LSID to the prefix http://zoobank.org/ . The LSID for this publication is: urn:lsid:zoobank.org:pub:7E91563A-395E-4B52-8AD1-009CFABBD472. The online version of this work is archived and available from the following digital repositories: PeerJ, PubMed Central and CLOCKSS.

## Results

Five tardigrade species from three genera were found in the present study: *Mil. inceptum* (Eutardigrada, Apochela), *Macrobiotus basiatus* sp. nov. (Eutardigrada, Parachela), and three species of Heterotardigrada, *Vir. miraviridis* sp. nov., *Vir. perviridis*, and *Vir. viridissimus*.

In terms of relative abundance, *Vir. viridissimus* was the most numerous species, followed by *Mil. inceptum*; both species were present on all six posts. The other three species were much less numerous with *Macrobiotus basiatus* sp. nov*.* found on four of the posts, whereas *Vir. perviridis* and *Vir. miraviridis* sp. nov. were present on only two of the posts.

### Taxonomic account

**Table:** 

**Phylum:** Tardigrada Doyère, 1840
**Class:** Heterotardigrada Marcus, 1927
**Family:** Echiniscidae Thulin, 1928
**Genus:***Viridiscus* Gąsiorek & Michalczyk, 2019, in Gąsiorek et al., 2019

**Figure 2:**
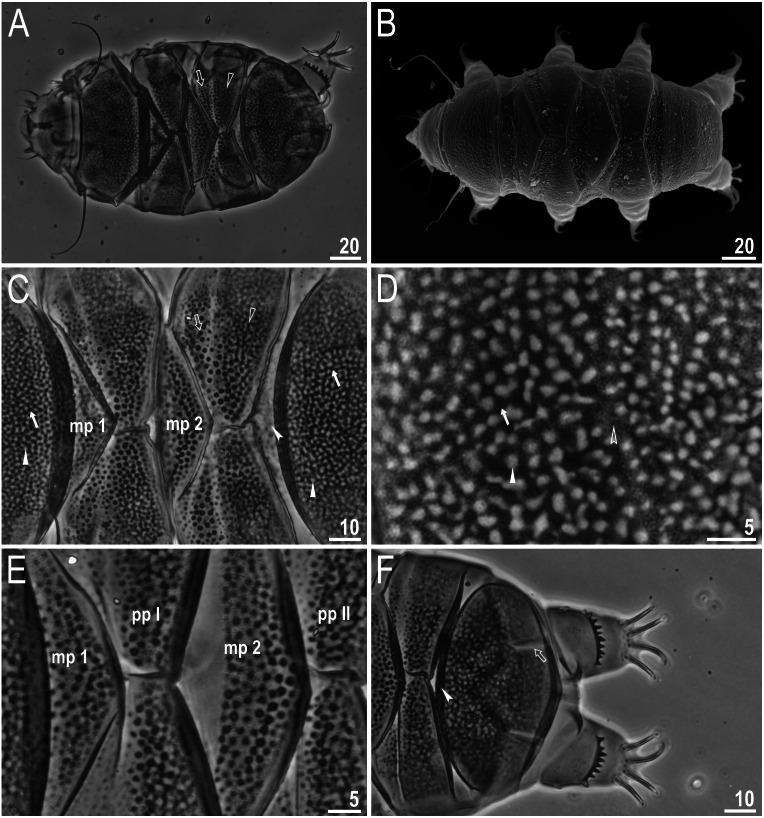
*Viridiscus miraviridis* sp. nov. (A) Habitus, dorsoventral assembled image, empty arrow indicates the anterior part of the paired plates, empty arrowhead indicates the posterior part of the paired plates (holotype, PCM). (B) Dorsal view (paratype, SEM). (C) Close up of the dorsal cuticle, empty arrow indicates the anterior part of the paired plates with large, dark unconnected granules, empty arrowhead indicates the posterior part of the paired plates with a mesh of irregular dark granules connected by dark lines, filled arrow indicates large, irregular dark granules, filled arrowhead indicates irregular light depressions between a mesh of dark granules, filled indented arrowhead indicates the absence of median plate 3, mp 1 and mp 2 indicate median plate 1 and 2, respectively (paratype, PCM). (D) Close up of the terminal plate, filled arrow indicates large, irregular dark granules, empty indented arrowhead indicates a dense background of fine dots, filled arrowhead indicates irregular light depressions between a mesh of dark granules (paratype, PCM). (E) Close up of the dorsal cuticle, mp 1 and mp 2 indicate median plate 1 and 2, respectively, pp I and pp II indicate paired plates I and II, respectively (paratype, PCM). (F) Terminal plate with two notches (empty arrow), filled indented arrowhead indicates the absence of median plate 3 (paratype, PCM). Scale bars in micrometres [µm]. Photo credit: Milena Roszkowska.

**Figure 3:**
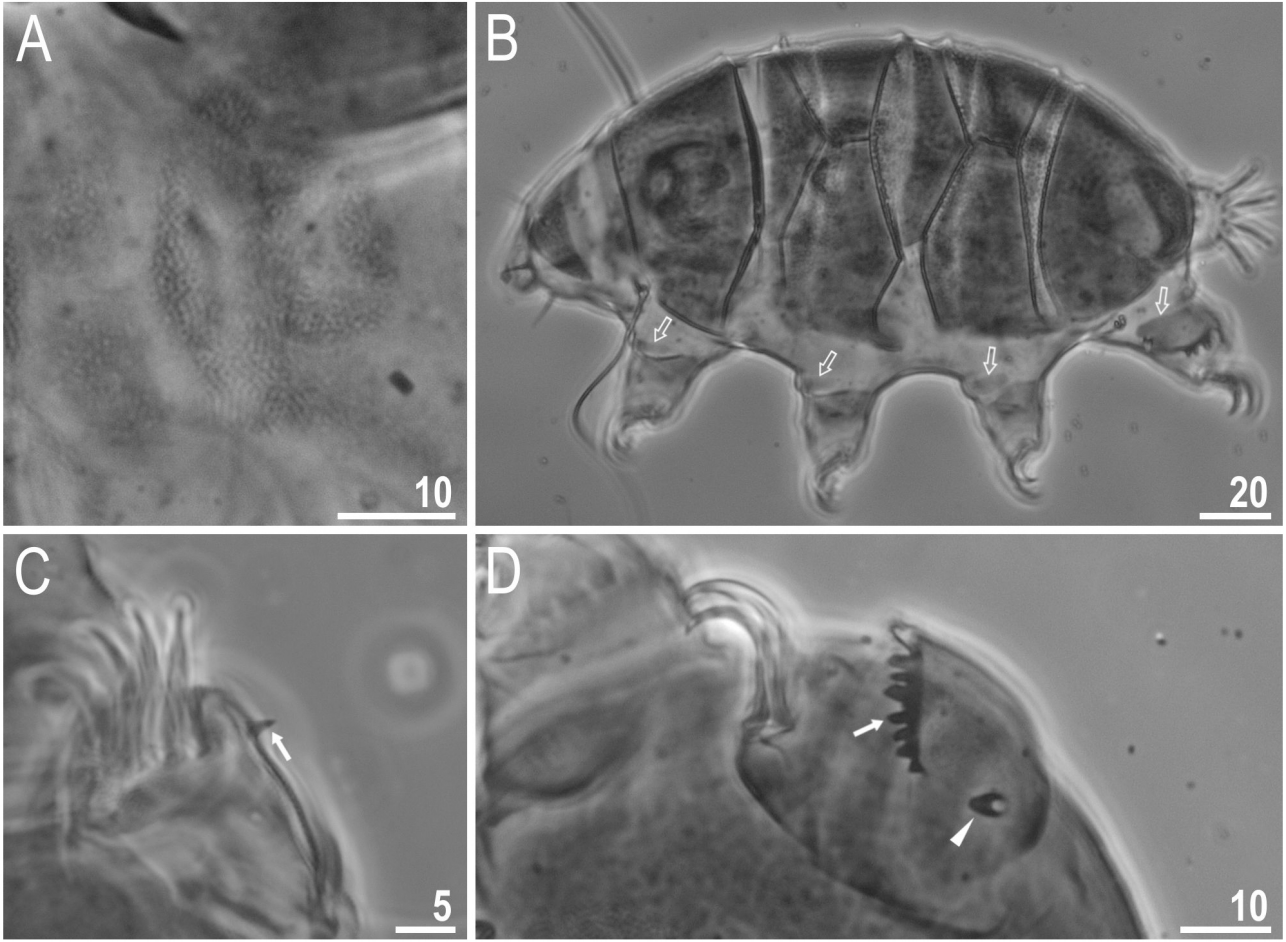
*Viridiscus miraviridis* sp. nov. (A) Ventral cuticle with tiny, regular granulation (paratype, PCM). (B) Animal in dorsolateral view, empty arrows indicate the narrow bands of granules on all legs (paratype, PCM). (C) Leg I, filled arrow indicates the spine on leg I (holotype, PCM). (D) Leg IV, filled arrowhead indicates the papilla on leg IV, filled arrow indicates the dentate collar (holotype, PCM). Scale bars in micrometres [µm]. Photo credit: Milena Roszkowska.

**Figure 4:**
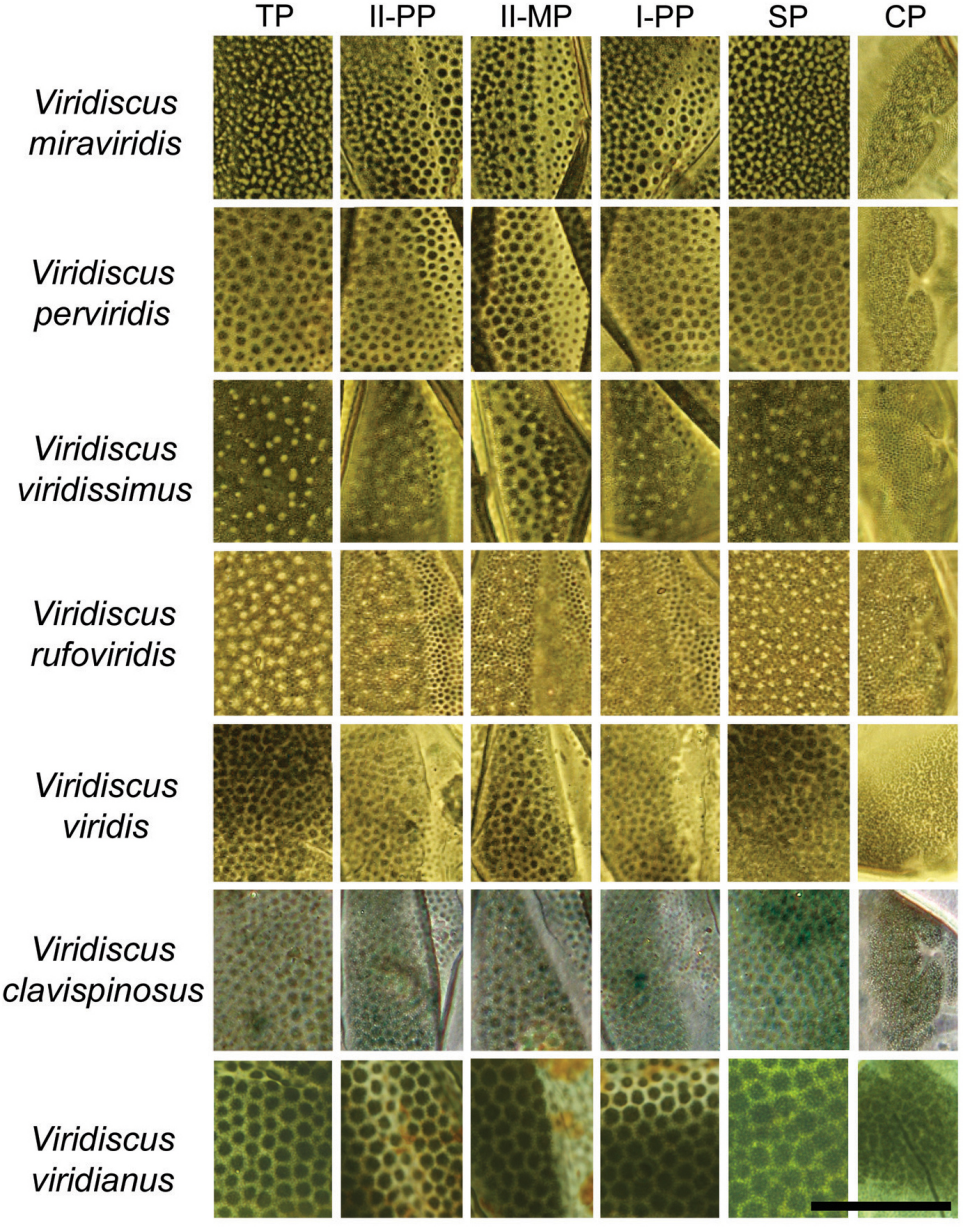
Comparison of the types of dorsal sculpture in the seven *Viridiscus* species (PCM). *Vir. miraviridis* sp. nov., *Vir. perviridis*, *Vir. viridissimus*, *Vir. rufoviridis*, *Vir. viridis*, *Vir. clavispinosus*, and *Vir. viridianus* are illustrated (green filter enhanced contrast/colour). The characters used in the comparison are: TP, terminal plate. II-PP, second paired plate. II-MP, second median plate. I-PP, first paired plate. SP, scapular plate. CP, cephalic plate. Scale bar = 20 µm. Photo credit: Roberto Guidetti, except *Vir. clavispinosus* by Giovanni Pilato.

***1. Viridiscus*** ***miraviridis*** **sp**. **nov.** *Genus name: urn:lsid:zoobank.org:act:2FCD58BD-5429-451A-BE2D-D6BA33C1ED31*, Species name: *urn:lsid:zoobank.org:act:0A9781C0-8F03-4A68-BE35-46DDB066E246. Publication LSID: urn:lsid:zoobank.org:pub:7E91563A-395E-4B52-8AD1-009CFABBD472.*

(Table 3, Figs. 2–4)

**Table 3:** Measurements and *sp* values of selected morphological structures of individuals of *Viridiscus miraviridis* sp. nov. mounted in Hoyer’s medium.

**CHARACTER**	**N**	**RANGE**		**MEAN**		**SD**		**Holotype**	
		*μ* m^a^	***sp***	*μ* m^a^	***sp***	*μ* m^a^	***sp***	*μ* m^a^	***sp***
Body length	30	133–247	*–*	200	*–*	29	*14*	191	*–*
Scapular plate length	30	30.1–53.3	–	43.8	*–*	6.2	*–*	42.7	*–*
Head appendages lengths			**		**		**		
Cirrus *internus*	30	8.9–17.8	*25.4–34.4*	13.5	*30.7*	2.3	*2.3*	13.5	*31.6*
Cephalic papilla	29	4.2–7.6	*12.4–15.9*	6.1	*14.0*	0.9	*0.8*	6.1	*14.3*
Cirrus *externus*	29	10.0–21.0	*30.5–40.6*	15.0	*34.4*	2.5	*2.3*	14.9	*34.9*
Clava	30	3.8–7.2	*12.6–16.4*	5.9	*13.6*	0.7	*0.9*	5.8	*13.6*
Cirrus *A*	27	31.2–65.4	*103.7–126.5*	50.9	*114.9*	7.8	*6.2*	50.3	*117.8*
Cirrus *A*/Body length ratio	27	21%–30%	–	25%	*–*	2%	*–*	26%	*–*
Cirrus *int*/*ext* length ratio	29	77%–96%	–	89%	*–*	4%	*–*	91%	*–*
Spine on leg I length	29	1.8–3.5	*5.5–7.1*	2.8	*6.3*	0.5	*0.4*	2.8	*6.6*
Papilla on leg IV length	23	3.0–4.7	*8.3–10.4*	4.0	*9.1*	0.4	*0.5*	3.9	*9.1*
Number of teeth on the collar	25	8–11	–	9.4	*–*	0.7	*–*	10	*–*
Notch length	24	11.4–19.3	*33.9–39.9*	16.1	*36.7*	2.1	*1.8*	16.6	*38.9*
Claw 1 lengths			**		**		**		
Branch	24	10.0–17.9	*29.9–35.6*	14.1	*32.3*	1.9	*1.6*	14.6	*34.2*
Spur length	10	1.1–1.9	*2.7–3.6*	1.4	*3.2*	0.3	*0.3*	1.3	*3.0*
Spur/branch length ratio	10	9%–11%	–	10%	*–*	1%	*–*	0	*–*
Claw 2 lengths			**		**		**		
Branch	24	9.0–15.4	*27.8–34.3*	13.1	*30.7*	1.7	*1.8*	13.2	*30.9*
Spur length	12	0.9–1.7	*2.9 – 4.2*	1.5	*3.3*	0.2	*0.4*	?	*?*
Spur/branch length ratio	12	9%–13%	–	11%	*–*	1%	*–*	?	*–*
Claw 3 lengths			**		**		**		
Branch	20	9.8–16.6	*27.9–33.5*	13.4	*30.5*	1.6	*1.6*	13.6	*31.9*
Spur length	9	1.2–1.5	*2.5 – 3.8*	1.4	*3.0*	0.1	*0.3*	1.3	*3.0*
Spur/branch length ratio	9	9%–12%	–	10%	*–*	1%	*–*	0	*–*
Claw 4 lengths			**		**		**		
Branch	25	11.4–19.7	*34.3–40.7*	16.2	*37.1*	2.1	*1.7*	15.1	*35.4*
Spur length	20	1.2–2.1	*3.2 – 4.7*	1.7	*3.9*	0.2	*0.4*	1.7	*4.0*
Spur/branch length ratio	20	9%–13%	*–*	11%	*–*	1%	*–*	0	*–*

**Notes.**

N number of specimens/structures measured, RANGE refers to the smallest and the largest structure among all measured specimens SD standard deviation ? trait oriented unsuitably for measurement *sp* ratio of the length of a given structure to the length of the scapular plate

a except for ratios, which are presented as percentages, and the number of teeth in dentate collar.

**Type locality:** 36°18′N, 82°22′W, 517 m asl; north side of the East Tennessee State University campus, Johnson City, Washington County, TN, USA, moss (*Grimmia* sp.) on concrete caps of brick fence posts; xerothermic habitat fully exposed to sun and wind.

**Etymology:** The species name is based on *mira* (L.) - wonderful, remarkable and *viridis* (L.) - green, which refers to the beautiful green color of the cuticle.

**Type material:** Holotype and 49 paratypes: 3 specimens of the 2nd instar; 20 specimens of adults) identified as females; and 27 individuals whose gender was not distinguished due to poor position on the slide or indistinct gonopore (i.e., as small, round circles that could not be definitely associated with a gender). No 1st instar.

**Type depositories:** The holotype and 9 paratypes are deposited in the National Museum of Natural History, Smithsonian Institution, Washington, DC, USA. (holotype and 4 paratypes, slide number USNM 1622558; 5 paratypes, slide number USNM 1622559). Additional paratypes are deposited in the Nelson Collection (28 slides), East Tennessee State University, Johnson City, TN, USA.; Bertolani Collection (2 slides with 7 specimens), University of Modena and Reggio Emilia, Modena, Italy; and Kaczmarek Collection (10 slides), Adam Mickiewicz University, Poznań, Poland.

### Description of the new species

#### Animals (measurements and statistics in Table 3)

**Females.** Body (Figs. 2A–2B) green, eyes absent or not visible after mounted in Hoyer’s. Except for the head appendages (internal and external cirri and drop-shaped cephalic papillae), only lateral cirrus *A* is present (with finger-like clava near the base) (Figs.  2A–2B). Gonopore rosette-shaped, anterior to the anus.

Cuticle on dorsal plates in general appears “two-layered,” constituted by a surface with shallow depressions over a uniform background of small granules (dark dots) (Figs.  2C–2F, 3). Head plate with very light mesh sculpture over a background of fine dots. On the scapular plate, terminal plate, and posterior parts of paired plates, there are large irregular dark granules and shallow depressions (in PCM) (Figs. 2C–2D, filled arrows) over a dense background of fine dots (Fig. 2D, empty indented arrowhead). These granules appear connected by dark lines, forming a lattice-like network interspersed with irregular light depressions (Figs. 2C–2D, filled arrowheads). Meshes are more irregular on the terminal plate (Fig. 2D). Dorsal plates are well developed. Head and scapular plates are not faceted. Paired plates I and II are divided into two parts, with anterior portion having a background of fine dots and large dark granules that are not connected (Figs. 2A, 2C, empty arrow), and the posterior portion with the sculpture as described above (Figs. 2A, 2C, empty arrowhead). Additionally, on lateral sides of the anterior parts of paired plates I and II, a small area without granules is present (but with background of fine dots). Median plates 1 and 2 are divided into two parts with different types of sculpture (Figs. 2C, 2E). Median plate 1 and 2 have cuticular pattern similar to the scapular plate, terminal plate, and posterior parts of paired plates. Median plate 3 is absent; instead there are dark, round granules over a very poorly visible background of fine dots (Figs. 2C, 2F, filled indented arrowhead). Terminal plate has two notches (Fig. 2F, empty arrow). Ventral plates are absent, but the cuticle has a tiny, regular granulation caused by dense cuticular pillars (Fig. 3A).

All legs have a narrow band of very fine granules (Fig. 3B, empty arrows). Dentate collar on legs IV has fine granulation and 8–14 teeth (teeth usually have only a single point and are slightly irregular) (Figs. 2F, 3D). Triangular spine on leg I (Fig. 3C, filled arrow) and finger-like papilla on leg IV are present (Fig. 3D, filled arrowhead). External claws of all legs are smooth, internal have spurs directed downwards; spurs are larger on the claws on the hind legs.

Males and eggs unknown.

### Differential diagnosis

Based on the specific morphology of the cuticle on the dorsal plates, the new species differs from all other species in the *viridis* group (Fig. 3). More specifically *Vir. miraviridis* sp. nov. differs from:

**1. *Viridiscus clavispinosus***, known only from its type locality in Republic of Cape Verde (Santo Antão Island off West Africa in the Atlantic Ocean) (Fontoura, Pilato & Lisi, 2011), by: different type of dorsal cuticle (in general dorsal granules appear connected by dark lines in PCM, forming a lattice-like network interspersed with irregular light depressions in the new species *vs* slightly raised dark tubercles that are not connected in *Vir. clavispinosus*); different shape of clava (finger-like clava in the new species *vs* spine-like clava in *Vir. clavispinosus*); area between paired plates II and terminal plate is sculptured *vs* smooth in *Vir. clavispinosus*; slightly smaller *sp* index of the clava (*12.6–16.4* in the new species *vs 16.5–18.1* in *Vir. clavispinosus*); larger *sp* index of cirrus *A* (*103.7–126.5* in the new species *vs 54.5–66.4* in *Vir. clavispinosus*); and larger cirrus *A*/body length ratio (21–30% in the new species *vs* 10.9–13.4% in *Vir. clavispinosus*).

**2. *Viridiscus perviridis***, known from Italy and the USA (Ramazzotti, 1959; Kaczmarek, Michalczyk & McInnes, 2016), by: different type of dorsal cuticle (in general dorsal granules appear connected by dark lines in PCM, forming a lattice-like network interspersed with irregular light depressions in the new species *vs* polygonal tubercles not connected in *Vir. perviridis*); and shorter cirrus *A* (31.2–65.4 µm in the new species *vs* 114.0–170.0 µm in *Vir. perviridis*).

**3. *Viridiscus rufoviridis***, known from Argentina and Brazil (Kaczmarek, Michalczyk & McInnes, 2015), by: different type of dorsal cuticle (in general dorsal granules appear connected by dark lines in PCM, forming a lattice-like network interspersed with irregular light depressions in the new species *vs* large dorsal granules absent in *Vir. rufoviridis*); entire body green (only caudal region of the body of green colour in *Vir. rufoviridis*); median plate 3 absent; terminal plate not faceted; and presence of spurs on internal claws.

**4. *Viridiscus viridianus*,** known from the Azores Islands (Portugal) and the USA (Pilato, Fontoura & Lisi, 2007), by: different type of dorsal cuticle (in general dorsal granules appear connected by dark lines in PCM, forming a lattice-like network interspersed with irregular light depressions in the new species *vs* polygonal tubercles not connected in *Vir. viridianus*); and cirrus *A* longer than scapular plate (cirrus *A* shorter than scapular plate in *Vir. viridianus*). *Viridiscus viridianus* from the Azores Islands was reported to be dark grey in colour (Fontoura, 1985), rather than green as in the species from the USA and the other species of *Viridiscus*.

**5. *Viridiscus viridis***, with confirmed localities only from the USA (Hawaiian Islands) (Pilato, Fontoura & Lisi, 2008), by: different type of dorsal cuticle (in general dorsal granules appear connected by dark lines in PCM, forming a lattice-like network interspersed with irregular light depressions in the new species *vs* polygonal tubercles not connected in *Vir. viridis*); longer cirrus *A* (31.2–65.4 µm in the new species *vs* 16.3–18.2 µm in *Vir. viridis*); larger *sp* index of cirrus *A* (*103.7–126.5* in the new species *vs 36.1–43.5* in *Vir. viridis*); and larger cirrus *A*/body length ratio (21–30% in the new species *vs* 7.4–9.6% in *Vir. viridis*).

**6. *Viridiscus viridissimus***, known from Romania, the USA and Venezuela (Péterfi, 1956; Kaczmarek, Michalczyk & McInnes, 2015; Kaczmarek, Michalczyk & McInnes, 2016), by: different type of dorsal cuticle (in general dorsal granules appear connected by dark lines in PCM, forming a lattice-like network interspersed with irregular light depressions in the new species *vs* large dorsal granules absent in *Vir. viridissimus*) and shorter cirrus *A* (cirrus *A*/body length ratio is 21–30% (31.2–65.4 µm) in the new species *vs* 36% (85.0 µm in specimen 234.0 µm long) in *Vir. viridissimus*).

***2. Viridiscus perviridis*** Ramazzotti, 1959

**Type locality:** Italy

**Material examined:** 137 specimens.

**Remarks:** *Viridiscus perviridis* has previously been reported from Italy and Tennessee (USA) (Ramazzotti, 1959; Maucci, 1987; McInnes, 1994; Nelson & Bartels, 2007; Kaczmarek, Michalczyk & McInnes, 2016).

No males were identified in this study. Based on the presence or absence of the anus and gonopore, the age structure of the population was as follows: 20, 1st instar; 22, 2nd instar; and 35, adults. For 60 individuals, 2nd instars and adults could not be distinguished.

***3. Viridiscus viridissimus*Péterfi, 1956**

**Type locality:** Romania

**Material examined:** 2,165 specimens.

**Remarks:** *Viridiscus viridissimus* has previously been reported from Europe (Romania), North America (Alabama, North Carolina, and Tennessee; USA) and South America (Venezuela) (Péterfi, 1956; Word, Folkerts & Mason, 1976; Nelson, 1979; Grigarick, Schuster & Nelson, 1983; Maucci, 1987; Nelson, Kincer & Williams, 1987; Dewel, Dewel & Nelson, 1992; Dewel, Dewel & Roush, 1992; McInnes, 1994; Dewel & Dewel, 1996; Nelson & Adkins, 2001; Dewel & Eibye-Jacobsen, 2006; Kaczmarek, Michalczyk & McInnes, 2015; Kaczmarek, Michalczyk & McInnes, 2016).

**Figure 5:**
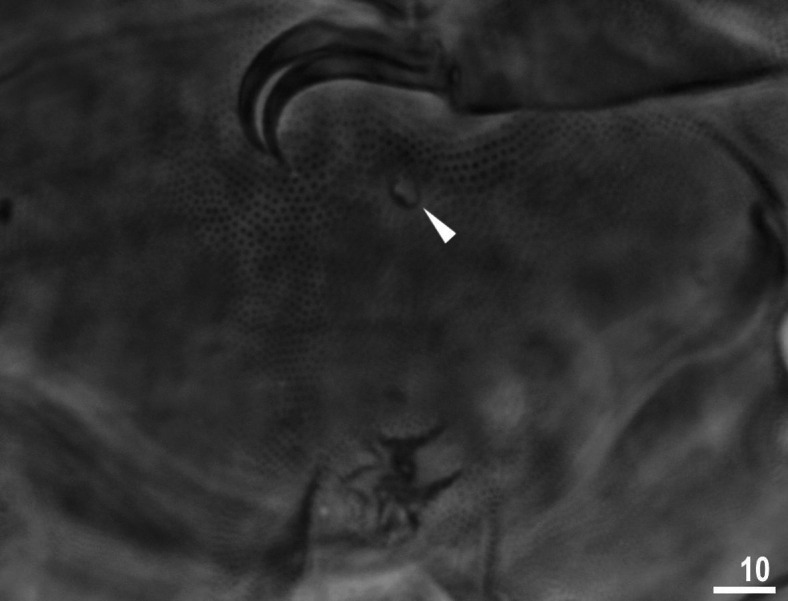
Male gonopore (filled arrowhead) of *Viridiscus viridissimus*. Scale bars in micrometres [µm]. Photo credit: Milena Roszkowska.

Males were present, but rare. The ratio of males to females for individuals whose gender could definitely be determined was 0.04 (29 males to 653 females). This is the first report of males in the genus *Viridiscus*. The male gonopore (Fig. 5) is oval, slightly raised, and located more posterior than the female gonopore, which has the typical rosette-shape.

The number of individuals that could be determined in each age class was as follows: 178, 1st instar; 299, 2nd instar; and 682, adults. Distinction between 2nd instar and adults could not be made for 1006 individuals due to the position of the gonopore (e.g., hidden by a leg) or due to the immaturity of the animal.

**Table:** 

**Class:** Eutardigrada Richters, 1926
**Order:** Apochela Schuster et al., 1980
**Family:** Milnesiidae Ramazzotti, 1962
**Genus:***Milnesium*Doyère, 1840

***4. Milnesium inceptum*Morek et al., 2019**

**Type locality:** Germany

**Material examined:** Total specimens: 1,164; sexually dimorphic males, 126.

**Remarks:** A pseudocryptic species, morphologically extremely similar to *Milnesium alpigenum* (Ehrenberg, 1853) but clearly different based on molecular data (Morek et al., 2019). This is a first record of this species from the USA. No males of *Mil. inceptum* were found by Morek et al. (2019) in populations from Bulgaria, Germany or Switzerland, and culturing of isolated virgin females confirmed that the type population is parthenogenetic. However, males were found to appear spontaneously in an otherwise parthenogenetic culture of the Japanese strain (Suzuki, 2008), suggesting that the species is facultatively parthenogenetic with males appearing only occasionally (Morek et al., 2019). Mature males have modified claws only on the first pair of legs, with the secondary (basal) branch of both claws shaped like a robust hook bearing a small spur (Rebecchi & Nelson, 1998).

#### DNA sequences

A good quality sequence for COI described here is accessible via GenBank accession number MT502117. Since the COI sequence was almost identical [only two nucleotide substitutions and p-distance 0.3% with sequence MK628723.1 (Morek et al., 2019)] to *Mil. inceptum*, we did not sequence 28S rRNA or ITS-2.

**Table:** 

**Class:** Eutardigrada Richters, 1926
**Order:** Parachela Schuster et al., 1980
**Superfamily:** Macrobiotoidea Thulin, 1928
**Family:** Macrobiotidae Thulin, 1928
**Genus:***Macrobiotus* C.A.S. Schultze, 1834

**Figure 6:**
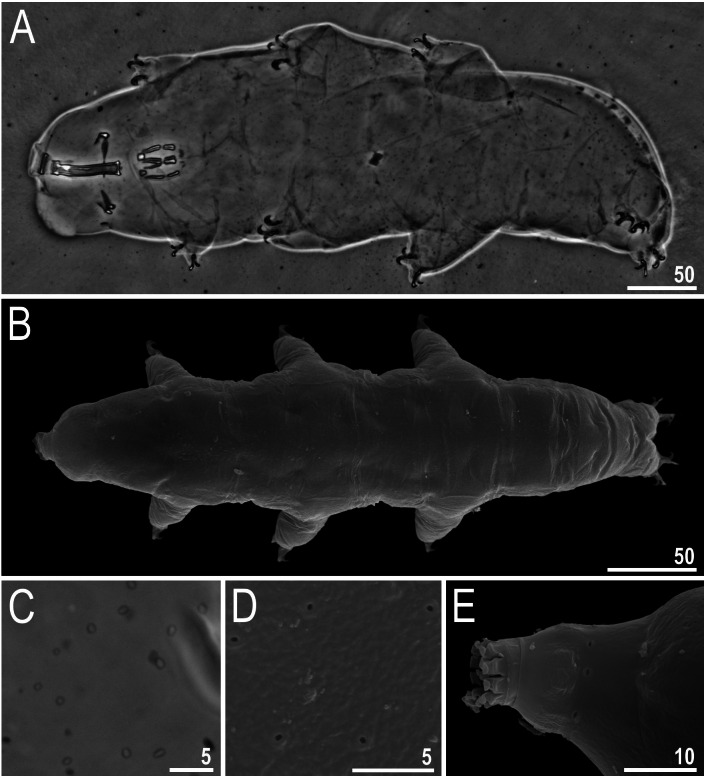
*Macrobiotus basiatus* sp. nov. (A) Habitus, dorsoventral assembled image (holotype, PCM). (B) Animal in dorsal view (paratype, SEM). (C) Cuticle covered with pores (holotype, PCM). (D) Cuticle covered with pores (paratype, SEM). (E) Ring of pores visible posterior to the mouth opening. Scale bars in micrometres [µm]. Photo credit: Milena Roszkowska.

**Figure 7:**
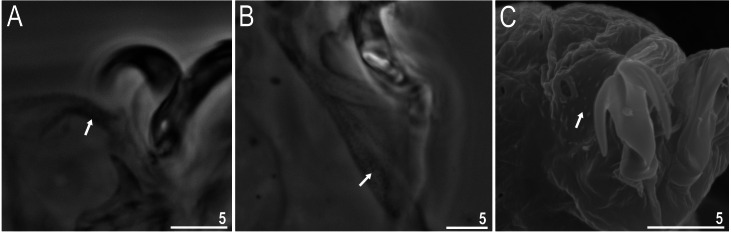
*Macrobiotus basiatus* sp. nov. granulation on legs (filled arrows). (A) Leg III (paratype, PCM). (B) Leg IV (paratype, PCM). (C) Leg IV (paratype, SEM). Scale bars in micrometres [µm]. Photo credit: Milena Roszkowska.

**Figure 8:**
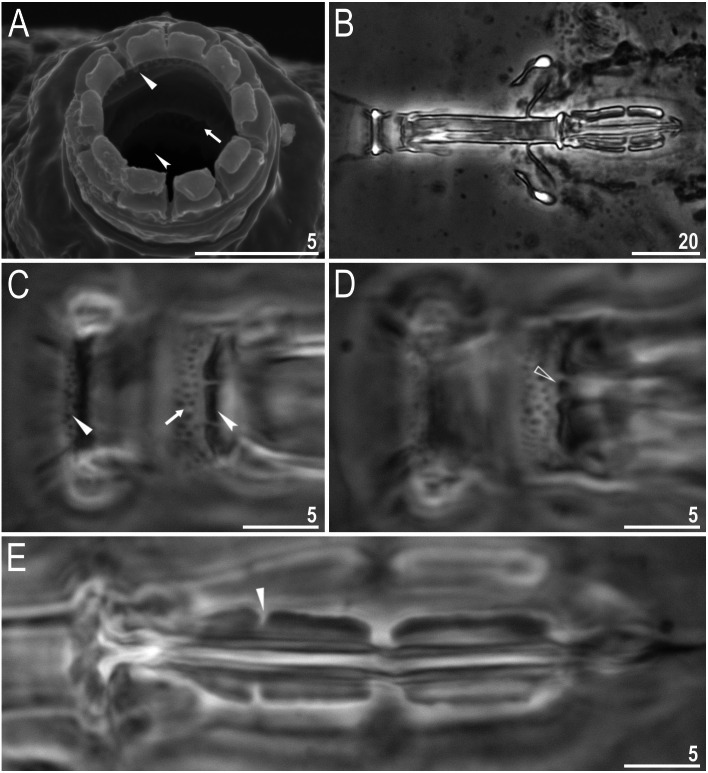
*Macrobiotus basiatus* sp. nov. claws. (A) Mouth opening with ten peribuccal lamellae, filled arrowhead indicates teeth of the first band, filled arrow indicates teeth of the second band and indented filled arrowhead indicates teeth of the third band (paratype, SEM). (B) Buccalpharyngeal apparatus (dorsoventral assembled image) (paratype, PCM). (C) Oral cavity armature (dorsal view), filled arrowhead indicates teeth of the first band, filled arrow indicates teeth of the second band and indented filled arrowhead indicates teeth of the third band. (D) Oral cavity armature (ventral view), empty arrowhead indicates median tooth of the third band on ventral side in shape of oval granule (paratype, PCM). (E) Macroplacoids, filled arrowhead indicates the first macroplacoid with a central constriction (paratype, PCM). Scale bars in micrometres [µm]. Photo credit: Milena Roszkowska.

**Figure 9:**
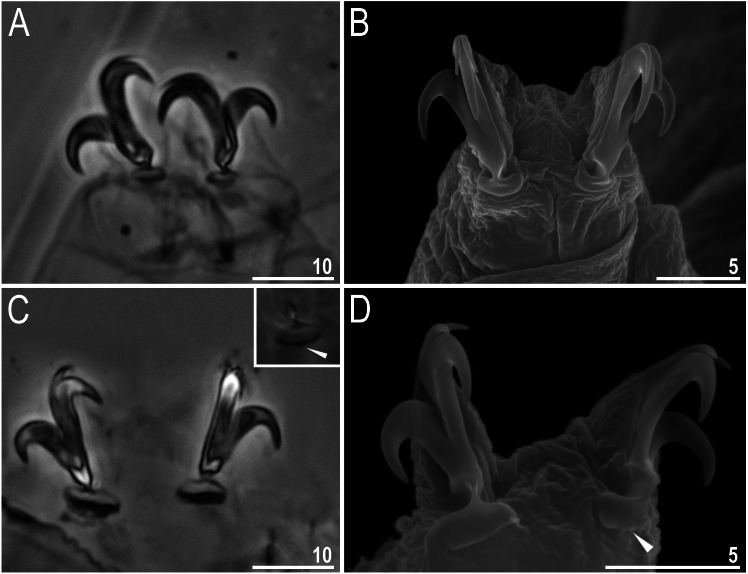
*Macrobiotus basiatus* sp. nov. claws. (A) Claws III with smooth lunules (paratype, PCM). (B) Claws II with smooth lunules (paratype, SEM). (C) Claws IV with smooth lunules, insert shows barely visible teeth found on one specimen (filled arrowhead) (paratype, PCM). (D) Claws IV with lunules with very tiny teeth visible (filled arrowhead) (paratype, SEM). Scale bars in micrometres [µm]. Photo credit: Milena Roszkowska.

**Figure 10:**
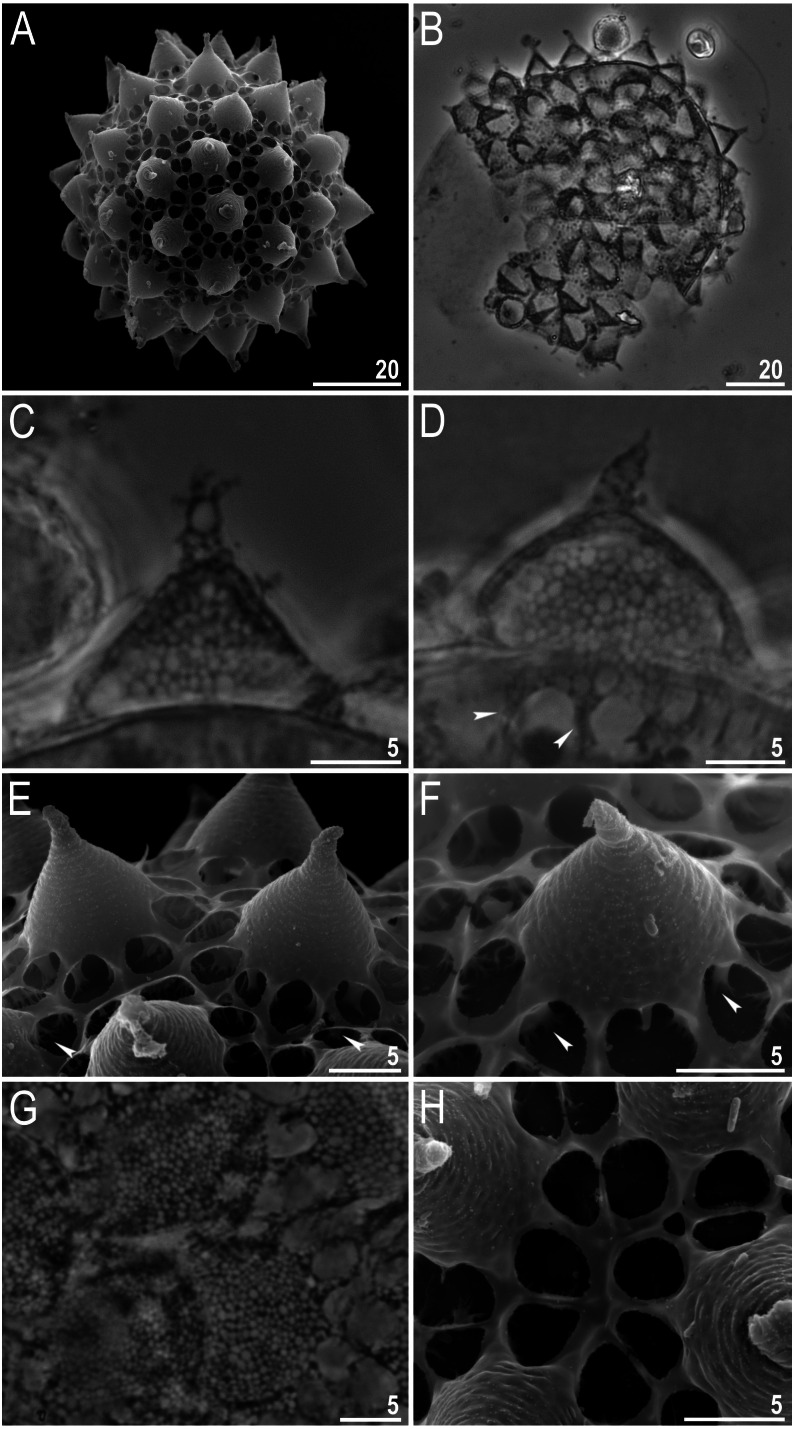
*Macrobiotus basiatus* sp. nov. eggs. (A) Entire egg (SEM). (B) Entire egg (PCM). (C–D) Egg processes, indented arrowheads indicate visible arms of areoles (PCM). (E–F) Egg processes, indented arrowheads indicate branched columns under processes and arms of areoles (SEM). (G) Egg surface with areoles (PCM). (H) Egg surface with areoles (SEM). Scale bars in micrometres [µm]. Photo credit: Milena Roszkowska.

***Macrobiotus basiatus***sp. nov****. *Genus name: urn:lsid:zoobank.org:act:42DFC178-D1BB-48EC-81F6-5B6BE3539AF2, Species name: urn:lsid:zoobank.org:act:95167BDA-B668-4D49-BFD2-1B314080F2F5. Publication LSID: urn:lsid:zoobank.org:pub:7E91563A-395E-4B52-8AD1-009CFABBD472.*

(Tables 4–5; Figs. 6–10)

**Table 4:** Measurements and *pt* values of selected morphological structures of individuals of *Macrobiotus basiatus* sp. nov. mounted in Hoyer’s medium.

**CHARACTER**	**N**	**RANGE**		**MEAN**		**SD**		**Holotype**	
		**µm**	***pt***	**µm**	***pt***	**µm**	***pt***	**µm**	***pt***
Body length	30	276–540	*–*	430	*–*	80	*–*	461	*–*
Buccalpharyngeal tube					**		**		
Buccal tube length	30	29.3–52.6	–	44.4	*–*	5.8	*–*	47.6	*–*
Stylet support insertion point	30	22.7–40.7	*74.4–78.9*	34.5	*77.8*	4.5	*1.1*	35.7	*75.0*
Buccal tube external width	30	4.6–10.4	*15.0–21.0*	8.1	*18.2*	1.5	*1.8*	7.3	*15.3*
Buccal tube internal width	28	3.5–8.0	*11.3–17.4*	6.3	*14.3*	1.2	*1.6*	5.8	*12.2*
Ventral lamina length	29	19.6–31.5	*55.7–63.7*	26.9	*60.0*	3.0	*2.0*	27.3	*57.4*
Placoid lengths					**		**		
Macroplacoid 1	30	7.3–17.5	*24.6–35.0*	13.2	*29.6*	2.6	*2.8*	13.6	*28.6*
Macroplacoid 2	30	4.8–11.0	*14.2 – 22.7*	8.2	*18.4*	1.7	*2.2*	8.5	*17.9*
Microplacoid	30	2.3–5.1	*6.6–10.3*	3.8	*8.6*	0.8	*1.0*	3.8	*8.0*
Macroplacoid row	30	13.5–30.9	*44.4–61.8*	23.6	*52.9*	4.4	*4.6*	25.0	*52.5*
Placoid row	28	17.2–37.8	*53.6–75.6*	28.9	*65.3*	5.4	*5.2*	30.2	*63.4*
Claw 1 lengths					**		**		
External primary branch	22	7.7–12.4	*21.4–26.3*	10.8	*24.5*	1.5	*1.2*	11.9	*25.0*
External secondary branch	18	6.0–10.3	*16.4–21.4*	8.3	*18.7*	1.2	*1.3*	9.5	*20.0*
Internal primary branch	22	7.3–11.5	*20.3–24.9*	10.1	*23.0*	1.4	*1.2*	11.0	*23.1*
Internal secondary branch	18	5.8–9.1	*15.3–18.9*	7.8	*17.3*	1.0	*1.0*	8.2	*17.2*
Claw 2 lengths					**		**		
External primary branch	26	7.9–13.4	*22.5–28.3*	11.4	*26.1*	1.8	*1.5*	13.2	*27.7*
External secondary branch	24	6.1–10.3	*16.2–21.6*	8.5	*19.6*	1.4	*1.2*	10.3	*21.6*
Internal primary branch	25	7.3–12.5	*20.7–26.7*	10.6	*24.2*	1.7	*1.4*	11.4	*23.9*
Internal secondary branch	22	5.4–9.7	*16.0–20.5*	8.0	*18.5*	1.3	*1.1*	9.4	*19.7*
Claw 3 lengths					**		**		
External primary branch	27	8.2–13.8	*22.7–29.1*	11.7	*26.2*	1.7	*1.5*	13.0	*27.3*
External secondary branch	27	6.5–11.1	*16.2–23.3*	8.9	*19.9*	1.3	*1.5*	11.1	*23.3*
Internal primary branch	25	8.0–13.0	*22.8–27.4*	10.9	*24.5*	1.5	*1.2*	12.5	*26.3*
Internal secondary branch	21	5.5–10.1	*16.3–21.3*	8.4	*18.8*	1.3	*1.4*	9.8	*20.6*
Claw 4 lengths					**		**		
Anterior primary branch	23	9.0–14.9	*21.8–31.0*	12.1	*27.2*	1.7	*1.8*	12.0	*25.2*
Anterior secondary branch	20	6.5–11.1	*17.9–23.4*	8.8	*19.9*	1.3	*1.2*	8.8	*18.5*
Posterior primary branch	22	9.1–14.8	*24.8–31.6*	12.7	*28.7*	1.7	*1.5*	13.5	*28.4*
Posterior secondary branch	21	6.4–11.2	*17.9–23.6*	9.3	*21.1*	1.4	*1.6*	10.4	*21.8*

**Notes.**

N number of specimens/structures measured, RANGE refers to the smallest and the largest structure among all measured specimens SD standard deviation *pt* ratio of the length of a given structure to the length of the buccal tube expressed as a percentage

**Type locality:** 36°18′N, 82°22′W, 517 m asl; north side of East Tennessee State University campus, Johnson City, Washington County, TN, USA, moss (*Grimmia* sp.) on concrete caps of brick fence posts; xerothermic habitat fully exposed to sun and wind.

**Etymology:** This species is named for the shape of the projections on the eggs, which resemble the famous chocolate candy, Hershey’s Kisses. From *basiatus* (L.), which means “kissed”

**Type material:** Holotype and 234 paratypes (199 specimens, 35 eggs –2 embryonated).

**Type depositories:** The holotype and 9 paratypes are deposited in the National Museum of Natural History, Smithsonian Institution, Washington, DC, USA (holotype slide number USNM 1622560; paratype slide numbers USNM 1622561, USNM 1622562, USNM 1622563, USNM 1622564, USNM 1622565 (2 specimens), USNM 1622566, USNM 1622567, USNM 1622568). Additional paratypes are deposited in the Nelson Collection (180 slides), East Tennessee State University, Johnson City, TN, USA; Bertolani Collection (12 slides with 20 animals and 2 eggs), University of Modena and Reggio Emilia, Modena, Italy; and Kaczmarek Collection (34 slides), Adam Mickiewicz University, Poznań, Poland.

**Table 5:** Measurements of selected morphological structures of eggs of *Macrobiotus basiatus* sp. nov.^a^

**CHARACTER**	**N**	**RANGE (***μ* m)	**MEAN (***μ* m)	**SD (***μ* m)
Diameter of egg without processes	8	72.3–88.9	82.2	6.6
Diameter of egg with processes	8	96.0–113.3	104.6	5.9
Process height	25	10.9–17.0	13.8	1.5
Process base width	25	14.2–19.0	16.4	1.2
Process base/height ratio	25	101%–142%	120%	12%
Distance between processes	23	3.9–7.5	5.3	0.9
Number of processes on the egg circumference	8	14–14	14.0	0.0

**Notes.**

a except for ratios, which are presented as percentages, and the number of processes on the egg circumference.

#### Description of the new species

##### Animals (measurements and statistics in Table 4)

Body colour white (transparent after fixation in Hoyer’s medium; Figs. 6A–6B). Larger specimens with several orange or brown irregular excretory granules. Eyes present in 97% of measured specimens; eyes were absent only in one specimen. Entire cuticle covered with conspicuous small, round pores (1.0–1.5 in diameter) distributed randomly but more clearly visible on the anterior part of the body (Figs. 6C–6E). Clearly visible granulation present on all legs, but much more indistinct on legs I-III (Figs. 7A–7C). Buccal-pharyngeal apparatus of the *Macrobiotus* type, with the ventral lamina and ten peribuccal lamellae (Figs. 8A–8B). Mouth antero-ventral. The oral cavity armature of the *hufelandi* type (Kaczmarek & Michalczyk, 2017), with three bands of teeth visible under PCM (Figs. 8C–8D). The first band of teeth composed of very small granules (PCM), arranged in a few rows and situated on the basal parts of the lamellae and just below them. The second band of teeth composed of small granules (PCM), arranged in a few rows, just above the third band of teeth. The third band of teeth composed of three dorsal and three ventral teeth in the shape of transverse ridges (PCM). Median tooth on ventral side in shape of an oval granule that can sometimes be divided into two parts. Pharyngeal bulb spherical with triangular apophyses, two rod-shaped macroplacoids and a thin microplacoid. Macroplacoid length configuration 2<1. The first macroplacoid with a central constriction (Fig. 8E). The second macroplacoid without constriction, but with latero-terminal globular projections. Claws of the *hufelandi* type, stout (Figs. 9A–9D). Primary branches with distinct accessory points. Lunules I–III smooth, but IV slightly indented, with very tiny teeth mainly visible with SEM (Figs. 9C insert and 9D). Thin paired bars under claws I–III present. Other cuticular thickenings on legs absent.

##### Eggs (measurements and statistics in Table 5)

Eggs spherical, ornamented and laid freely (Figs. 10A–10B). Processes conical with short and sometimes a slightly flexible elongated tip (Figs. 10A–10F). In PCM, the surface of the processes and arms of the areoles have a reticular pattern with a regular rounded mesh (Figs. 10C–10D). In SEM, the processes and arms of areoles are smooth and visibly raised above the internal surface of the egg and connected with it by branched columns (Figs. 10E– 10F). Each process is surrounded by 9–10 areoles, smooth inside (Figs. 10D–10H), not always visible in PCM (Fig. 10B). The number of areoles between two neighboring processes is always two (Fig. 10H).

#### Differential diagnosis

The new species, based on the morphology of the eggs and adults, is most similar to *Macrobiotus deceptor* Meyer et al., 2017, *Macrobiotus nelsonae* Guidetti, 1998, *Macrobiotus pallarii* Maucci, 1954, and *Macrobiotus ragonesei* Binda et al., 2001, but it differs specifically from:

1. *Macrobiotus deceptor*, known only from Saint Mary Parish, Antigua (Meyer et al., 2017), by: presence of eyes, cuticular pores, and granulation on legs in the new species, oval areoles around egg processes (polygonal in *Mac. deceptor*), shorter egg processes (10.9–17.0 µm in the new species *vs* 21.2–30.3 µm in *Mac. deceptor*) and smaller number of areoles around egg processes (*ca.* 8 in the new species *vs* 10–12 in *Mac. deceptor*).
2. *Macrobiotus nelsonae*, known only from Roan Mountain, Tennessee, USA (Guidetti, 1998), by: the stylet supports situated in more anterior position (*pt 74.4–78.9* in new species *vs 79.4–84.5* in *Mac. nelsonae*), shorter egg processes (10.9–17.0 µm in new species *vs* 20.8–31.7 µm in *Mac. nelsonae*), oval areoles around egg processes in the new species (polygonal in *Mac. nelsonae*), narrower egg processes (14.2–19.0 µm in new species *vs* 20.0–34.7 µm in *Mac. nelsonae*) and smaller number of areoles around egg processes (*ca.* 8 in new species *vs* 11–12 in *Mac. nelsonae*).
3. *Macrobiotus pallarii*, reported from Asia, Europe and North America (McInnes, 1994), by: the presence of slightly indented with very tiny teeth on lunules (visible with SEM) on legs IV (smooth lunules in *Macrobiotus pallarii*) and presence of two rows of areoles around each egg processes (only one row of areoles in *Mac. pallarii*). *Macrobiotus pallarii*, is poorly described and needs revision, but we have included it here for comparison with the new species.
4. *Macrobiotus ragonesei*, reported from North Kivu Province, Democratic Republic of the Congo (Binda et al., 2001), by: the presence of granulation on legs I–III, the presence of regular reticular mesh on the entire surface of the egg processes (egg processes with only the basal part covered with regular reticular mesh in *Mac. ragonesei*), higher number of processes on the egg circumference (14 in the new species *vs* 10–12 in *Mac. ragonesei*), smaller eggs (72.3–88.9 µm and 96.0–113.3 µm with and without processes, respectively, in the new species *vs* 92.0–100.0 µm and 126.0–136.0 µm with and without processes, respectively, in *Mac. ragonesei*), shorter egg processes (10.9–17.0 µm in the new species *vs* 20.0–23.0 µm in *Mac. ragonesei*), and narrower egg processes (14.2–19.0 µm in the new species *vs* 20.0–26.0 µm in *Mac. ragonesei*).

##### DNA sequences

Good quality sequences described here are accessible via GenBank accession numbers MT488397, MT488398, MT488399, MT488400, MT488401, MT488402 for 28S rRNA, MT498094 for 18S rRNA, MT505165, MT505166, MT505167, MT505168, MT505169 and MT505170 for ITS-2, and MT502116 for COI. We obtained two exoskeletons of *Mac. basiatus* sp. nov. after DNA isolation, which are deposited in the Kaczmarek collection, labelled USA/NEL/4/S and USA/NEL/6/S.

#### Molecular differential diagnosis

All the obtained DNA barcode sequences of *Mac*. *basiatus* sp. nov. were unique and distinct from those deposited in GenBank.

The p-distances between new species and other deposited in GenBank are:

1. 18S rRNA: **0.4–3.6% (1.9% on average)**, with the most similar being the *Mac. hufelandi* group (FJ435738 and FJ435739) (Guil & Giribet, 2012) and the least similar being *Macrobiotus polypiformis* Roszkowska et al., 2017 (KX810008) (Roszkowska et al., 2017).
2. 28S rRNA: **1.8–11.8% (6.6% on average)**, with the most similar being *Mac. hufelandi* group (FJ435751 and FJ435754 –FJ435755) (Guil & Giribet, 2012) and the least similar being *Mac. polypiformis* (KX810009) (Roszkowska et al., 2017).
3. COI: **15.3–23.4% (19.1% on average)**, with the most similar being *Macrobiotus terminalis* Bertolani & Rebecchi, 1993 (AY598775) (Guidetti et al., 2005) and the least similar being *Mac. polypiformis* (KX810012) (Roszkowska et al., 2017).
4. ITS-2: **0.5–30.6% (17.2% on average)**, with the most similar being *Macrobiotus canaricus* (Stec, Krzywański & Michalczyk, 2018) (MH063929) (Stec, Krzywański & Michalczyk, 2018) and the least similar being *Macrobiotus scoticus* Stec et al., 2017 (KY797268) (Stec et al., 2017).

### Discussion

The most numerous species, *Vir. viridissimus*, was obviously a well-established species on the fence posts. With the second highest relative abundance, *Mil. inceptum* was also considered highly successful in this xerothermic habitat. Previously found in samples from this site (Nelson & Adkins, 2001) but undescribed until now, *Mac. basiatus* sp. nov. was relatively low in abundance.

The large number of *Mil. inceptum* individuals is interesting since this species is a predator. If *Milnesium* individuals fed on *Viridiscus* or *Macrobiotus* specimens, then cuticular parts of the animals (e.g., claws, buccal tubes, dorsal plates) should have been visible within the gut, however, no evidence of this was found. Thus, the major food supply for such a large population of this predator is probably constituted by the numerous rotifers and nematodes, verified by the presence of rotifer jaws in guts of some *Mil. inceptum* specimens.

The genus *Viridiscus* was recently established by Gąsiorek & Michalczyk in Gąsiorek et al. (2019) for the *viridis* group of species previously included in the genus *Echiniscus*. *Viridiscus* is characterized by species with a fully or partially green cuticle (although sometimes brown, gray, or almost black), a dorsal plate sculpture composed of an intracuticular foam-like layer and small, flat and densely arranged epicuticular granules, no cuticular appendages other than cephalic appendages and lateral cirri *A*, well-developed sabre-like claws, absence of males, and inhabiting terrestrial ecosystems. The genus includes *Vir. clavispinosus*, *Vir. perviridis*, *Vir. rufoviridis*, *Vir. viridis*, *Vir. viridianus*, *Vir. viridissimus*, and now *Vir. miraviridis* sp. nov. The dorsal cuticle is characterized by an evident surface (with “pits”, granules, meshes, etc.) characteristic of each species, overlying a quite uniform background of fine dots. In *Viridiscus* a ventral cuticular pattern is also present, but it is also present in species from different heterotardigrade genera; this character has not yet been evaluated for its taxonomic value in the genus. The presence of a green cuticle, a primary morphological criterion for *Viridiscus*, is also found in *Echiniscus pooensis* Rodriguez-Roda, 1948, which has lateral and dorsal spines on the cuticular plates and therefore was not included in the genus in Gąsiorek et al. (2019).

We noted also that the specimens of *Vir. rufoviridis* of the Maucci collection present a different structure of the dorsal cuticle of the paired plates and second median plate than any other species of *Viridiscus*, which was also reported by Pilato, Fontoura & Lisi (2008). In fact, the granules present in the anterior position of these plates are not overlying the background of fine dots, but these granules become smaller (around the middle portion of each plate) forming the background of small dots present under the posterior surface of the plates characterized by white “pits.”

Males are very rare in the genera derived from the evolutionary line of the former *Echiniscus* “*arctomys* group” according to Gąsiorek et al. (2019). Only 37 years ago, males were considered completely absent in the widespread genus *Echiniscus*, at that time composed of about 112 species (Ramazzotti & Maucci, 1983), including the former *viridis* group (now the genus *Viridiscus*) within the “*arctomys* group”. The first report of males in *Echiniscus* species was published by Dastych (1987) and later by others (e.g., Claxton, 1996; Miller, Claxton & Heatwole, 1999; Mitchell & Romano III, 2007; Claxton & Dastych, 2017, who reported males in several species of *Echiniscus*. Miller, Claxton & Heatwole (1999) hypothesized a Gondwana origin of bisexual *Echiniscus* species, comprising those of the “*arctomys* group.” Although Mitchell & Romano III (2007) reported the first males in the former *Echiniscus mauccii* [now *Claxtonia mauccii* (Ramazzotti, 1956)] in North America, our finding of males in *Vir*. *miraviridis* sp. nov. confirms the presence of males in the genus *Viridiscus* on the continent. Therefore, the proposal of a Gondwana origin of bisexual species in this evolutionary line needs to be re-evaluated. In the description of the new genus *Viridiscus* in Gąsiorek et al. (2019), the species in the genus were reported to reproduce by parthenogenesis. However, in our study, males of *Vir. viridissimus* were rare (0.04%) but present, raising questions about that one aspect of the generic description. Additional observations of gonopores may reveal rare males in other species of *Viridiscus*.

The distribution of the genus *Viridiscus* is global, occurring throughout Europe, North and South America and on Pacific and Atlantic islands, leading to the hypothesis that the genus is also present in areas not yet well investigated. Although *Vir. viridis* has been widely reported from Europe, Hawaii (Oahu Is.) USA, North and South America, at present only the Hawaiian reports are correct according to Pilato, Fontoura & Lisi (2008). A similar wide range (Europe, North and South America) has been reported for *Vir. viridissimus*, but probably this species also has a narrower distribution. *Viridiscus perviridis* is reported from Europe and North America, *Vir. viridianus* from North America and the Azores Islands (Atlantic Ocean), and *Vir. rufoviridis* only from South America. *Viridiscus clavispinosus* is currently considered endemic to Santo Antão Island (Republic of Cape Verde, Atlantic Ocean, off West Africa) and *Viridiscus miraviridis* sp. nov. is known only from Tennessee (USA, North America) (McInnes, 1994; Fontoura, Pilato & Lisi, 2011; Kaczmarek, Michalczyk & McInnes, 2015; Kaczmarek, Michalczyk & McInnes, 2016). The records of the widely distributed species need further confirmation.

### Conclusions

Five species of tardigrades, including two species new to science, were found in moss from a xerothermic habitat in Tennessee: *Milnesium inceptum* (Eutardigrada), *Macrobiotus basiatus* sp. nov. (Eutardigrada), and three species of Heterotardigrada, *Viridiscus. miraviridis* sp. nov., *Vir. perviridis*, and *Vir. viridissimus*. This is the first record of *Mil. inceptum* from North America, confirmed by COI analysis, and the first record of males in the genus *Viridiscus*. The two new species were identified by an integrative analysis of morphological and/or molecular characters. This research increases our knowledge of tardigrades in Tennessee and North America and adds to the database on biogeography and distribution. Further investigations could examine the tardigrade community structure, analysing the co-existence of multiple *Viridiscus* species in the isolated habitats, as well as determining the chemical composition of the green pigment in the *Viridiscus* cuticle, as little is known of the chemical composition of various pigments in tardigrades.

##  Supplemental Information

10.7717/peerj.10251/supp-1 Supplemental Information 1 Uncorrected pairwise distances

10.7717/peerj.10251/supp-2 Supplemental Information 2 ** Measurements of data of* Viridiscus miraviridis* sp nov Raw Data

10.7717/peerj.10251/supp-3 Supplemental Information 3 Measurements of characters in *Macrobiotus basciatus* sp. nov

10.7717/peerj.10251/supp-4 Supplemental Information 4 Sequences and GenBank numbers for 18S rRNA, 28S rRNa, ITS2, and COI for Macrobiotus basiatus n sp. and COI for Milnesium tardigradum

10.7717/peerj.10251/supp-5 Supplemental Information 5 Sequences of 18S rRNA for Macrobiotus basiatus n. sp

10.7717/peerj.10251/supp-6 Supplemental Information 6 Sequences of COI for Macrobiotus basiatus n. sp

10.7717/peerj.10251/supp-7 Supplemental Information 7 Sequences of ITS2 for Macrobiotus basiatus n. sp

10.7717/peerj.10251/supp-8 Supplemental Information 8 Sequences of 28S rRNA for Macrobiotus basiatus n. sp

## Additional Information and Declarations

### DNA Deposition

The following information was supplied regarding the deposition of DNA sequences: The *Macrobiotus basiatus* sequences are available at Genbank: MT488397, MT488398, MT488399, MT488400, MT488401, MT488402 for 28S rRNA, MT498094 for 18S rRNA, MT505165, MT505166, MT505167, MT505168, MT505169 and MT505170 for ITS-2, and MT502116 for COI. The *Mil. inceptum* sequence for COI is available at Genbank: MT502117. The sequences are also available in the Supplementary Files.

### Data Availability

The following information was supplied regarding data availability: The raw measurements for *Mac. basciatus* and for *Vir. miraviridis* and the sequences are available in the Supplementary Files. The specimens are deposited at the National Museum of Natural History: USNM 1622558, USNM 1622559, USNM 1622560, USNM 1622561, USNM 1622562, USNM 1622563, USNM 1622564, USNM 1622565, USNM 1622566, USNM 1622567, USNM 1622568.

### New Species Registration

The following information was supplied regarding the registration of a newly described species: Publication LSID: urn:lsid:zoobank.org:pub:7E91563A-395E-4B52-8AD1-009CFABBD472. Genus names: *Viridiscus miraviridis* sp. nov.: urn:lsid:zoobank.org:act:0A9781C0-8F03-4A68-BE35-46DDB066E246 *Macrobiotus basiatus* sp. nov.: urn:lsid:zoobank.org:act:95167BDA-B668-4D49-BFD2-1B314080F2F5 Species names: *Viridiscus miraviridis* sp. nov. LSID: urn:lsid:zoobank.org:act:0A9781C0-8F03-4A68-BE35-46DDB066E246. *Macrobiotus basiatus* sp. nov. LSID: urn:lsid:zoobank.org:act:95167BDA-B668-4D49-BFD2-1B314080F2F5.
