# Supplementary material for: Two new species of Tardigrada from moss cushions (Grimmia sp.) in a xerothermic habitat in northeast Tennessee (USA, North America), with the first identification of males in the genus Viridiscus
Source: PeerJ. 2020 Nov 23;8:e10251. doi: 10.7717/peerj.10251 (PMC7690296; doi:10.7717/peerj.10251)
Supplement: Supplemental Information 4 [file peerj-08-10251-s004.docx]

| Sequence | Name | **Gen Bank accession number** | Sequence |
| --- | --- | --- | --- |
| 28S rRNA | >Seq1 [organism=Macrobiotus basiatus] [isolate=USA/NEL/1] 28S ribosomal RNA gene, partial sequence | **MT488397** | TAAGCATATTACTAAGCGGAGGAAAAGAAACCAACGGGGATGCCGATAGTAACTGCGAGTGAAATCGGCCAAGCCCAGCGCCGAATCCTATTGCTGGCAACGGTGACGGGAACTGTGGCGTGAAGAACGCTCTTACCGGTACGGTTCGTGTGCGTAAGTTCTCCTGAGTGAGGCTCCATCCCAGTGAGGGTGCAAGGCCCGTATCGCGCACAACTGGTACCGGTGTAAGTGTTCGGAGAGTCGCCTTGTTTGTGAGTACAAGGTGAAGTCGGTGGTAAACTCCATCGAAGGCTAAATATGACCACGAGTCCGATAGCGAACAAGTACCGTGAGGGAAAATTGAAAAGCACTTTGAAGAGAGAGCGAAACAGTGCGTGAAACCGCTCAGAGGCAAGCAGATGGGGCCTCGAAGGCAAAGCAGCGAATTCAGCTGGTGGTCTGCGTGGCCGGCCGGTGTAGGGATCTTAACGACTCTTACCGGTTGTGTTCTGCGTAGGTGCCAGTGCACTTTCGTTGTTTGTACGCCACTGCCGTTGAGTGAGCATCCGTCGGGTTGACGTGTGAAGCCTTACTCCTTCACGGGCGTAGGTGCTTACAGTTGACTTTGTACGCGTTTGCACCTCAACCGGTCATGTCAGCATGTGCCAGCGTTTGCGTTGGGCCGGTGCGCTCTGTCGGTTGTCGTGGGATGACGAGCTTGCTCGGCTC |
|  | >Seq2 [organism=Macrobiotus basiatus] [isolate=USA/NEL/2] 28S ribosomal RNA gene, partial sequence | **MT488398** | TAAGCATATTANTAAGCGGAGGAAAAGAAACCAACGGGGATGCCGATAGTAACTGCGAGTGAAATCGGCCAAGCCCAGCGCCGAATCCTATTGCTGGCAACGGTGACGGGAACTGTGGCGTGAAGAACGCTCTTACCGGTACGGTTCGTGTGCGTAAGTTCTCCTGAGTGAGGCTCCATCCCAGTGAGGGTGCAAGGCCCGTATCGCGCACAACTGGTACCGGTGTAAGTGTTCGGAGAGTCGCCTTGTTTGTGAGTACAAGGTGAAGTCGGTGGTAAACTCCATCGAAGGCTAAATATGACCACGAGTCCGATAGCGAACAAGTACCGTGAGGGAAAATTGAAAAGCACTTTGAAGAGAGAGCGAAACAGTGCGTGAAACCGCTCAGAGGCAAGCAGATGGGGCCTCGAAGGCAAAGCAGCGAATTCAGCTGGTGGTCTGCGTGGCCGGCCGGTGTAGGGATCTTAACGACTCTTACCGGTTGTGTTCTGCGTAGGTGCCAGTGCACTTTCGTTGTTTGTACGCCACTGCCGTTGAGTGAGCATCCGTCGGGTTGACGTGTGAAGCCTTACTCCTTCACGGGCGTAGGTGCTTACAGTTGACTTTGTACGCGTTTGCACCTCAACCGGTCATGTCAGCATGTGCCAGCGTTTGCGTTGGGCCGGTGCGCTCTGTCGGTTGTCGTGGGATGACGAGCTTGCTCGGCTCCTCGATGATTAGTAGAATCGTGCTGGTTTTCAACGTCGGTACATTGTAGATTCGGTGGCGAGTAGACGGCTGCCCATCTAACCCGTCTTGAA |
|  | >Seq3 [organism=Macrobiotus basiatus] [isolate=USA/NEL/3] 28S ribosomal RNA gene, partial sequence | **MT488399** | TAAGCATATTACTAAGCGGAGGAAAAGAAACCAACGGGGATGCCGATAGTAACTGCGAGTGAAATCGGCCAAGCCCAGCGCCGAATCCTATTGCTGGCAACGGTGACGGGAACTGTGGCGTGAAGAACGCTCTTACCGGTACGGTTCGTGTGCGTAAGTTCTCCTGAGTGAGGCTCCATCCCAGTGAGGGTGCAAGGCCCGTATCGCGCACAACTGGTACCGGTGTAAGTGTTCGGAGAGTCGCCTTGTTTGTGAGTACAAGGTGAAGTCGGTGGTAAACTCCATCGAAGGCTAAATATGACCACGAGTCCGATAGCGAACAAGTACCGTGAGGGAAAATTGAAAAGCACTTTGAAGAGAGAGCGAAACAGTGCGTGAAACCGCTCAGAGGCAAGCAGATGGGGCCTCGAAGGCAAAGCAGCGAATTCAGCTGGTGGTCTGCGTGGCCGGCCGGTGTAGGGATCTTAACGACTCTTACCGGTTGTGTTCTGCGTAGGTGCCAGTGCACTTTCGTTGTTTGTACGCCACTGCCGTTGAGTGAGCATCCGTCGGGTTGACGTGTGAAGCCTTACTCCTTCACGGGCGTAGGTGCTTACAGTTGACTTTGTACGCGTTTGCACCTCAACCGGTCATGTCAGCATGTGCCAGCGTTTGCGTTGGGCCGGTGCGCTCTGTCGGTTGTCGTGGGATGACGAGCTTGCTCGGCTCCTCGATGATTAGTAGAATCGTGCTGGTTTTCAACGTCGGTACATTGTAGATTCGGTGGCGAGTAGACGGCTGCCCATCTAACCCGTCTTGAAAC |
|  | >Seq4 [organism=Macrobiotus basiatus] [isolate=USA/NEL/4] 28S ribosomal RNA gene, partial sequence | **MT488400** | TTAAGCATATTACTAAGCGGAGGAAAAGAAACCAACGGGGATGCCGATAGTAACTGCGAGTGAAATCGGCCAAGCCCAGCGCCGAATCCTATTGCTGGCAACGGTGACGGGAACTGTGGCGTGAAGAACGCTCTTACCGGTACGGTTCGTGTGCGTAAGTTCTCCTGAGTGAGGCTCCATCCCAGTGAGGGTGCAAGGCCCGTATCGCGCACAACTGGTACCGGTGTAAGTGTTCGGAGAGTCGCCTTGTTTGTGAGTACAAGGTGAAGTCGGTGGTAAACTCCATCGAAGGCTAAATATGACCACGAGTCCGATAGCGAACAAGTACCGTGAGGGAAAATTGAAAAGCACTTTGAAGAGAGAGCGAAACAGTGCGTGAAACCGCTCAGAGGCAAGCAGATGGGGCCTCGAAGGCAAAGCAGCGAATTCAGCTGGTGGTCTGCGTGGCCGGCCGGTGTAGGGATCTTAACGACTCTTACCGGTTGTGTTCTGCGTAGGTGCCAGTGCACTTTCGTTGTTTGTACGCCACTGCCGTTGAGTGAGCATCCGTCGGGTTGACGTGTGAAGCCTTACTCCTTCACGGGCGTAGGTGCTTACAGTTGACTTTGTACGCGTTTGCACCTCAACCGGTCATGTCAGCATGTGCCAGCGTTTGCGTTGGGCCGGTGCGCTCTGTCGGTTGTCGTGGGATGACGAGCTTGCTCGGCTCCTCGATGATTAGTAGAATCGTGCTGGTTTTCAACGTCGGTACATTGTAGATTCGGTGGSGAGNANACGGNTGCCCATCTAACCC |
|  | >Seq5 [organism=Macrobiotus basiatus] [isolate=USA/NEL/5] 28S ribosomal RNA gene, partial sequence | **MT488401** | TNAGCATATTANTAAGCGGAGGAAAAGAAACCAACGGGGATGCCGATAGTAACTGCGAGTGAAATCGGCCAAGCCCAGCGCCGAATCCTATTGCTGGCAACGGTGACGGGAACTGTGGCGTGAAGAACGCTCTTACCGGTACGGTTCGTGTGCGTAAGTTCTCCTGAGTGAGGCTCCATCCCAGTGAGGGTGCAAGGCCCGTATCGCGCACAACTGGTACCGGTGTAAGTGTTCGGAGAGTCGCCTTGTTTGTGAGTACAAGGTGAAGTCGGTGGTAAACTCCATCGAAGGCTAAATATGACCACGAGTCCGATAGCGAACAAGTACCGTGAGGGAAAATTGAAAAGCACTTTGAAGAGAGAGCGAAACAGTGCGTGAAACCGCTCAGAGGCAAGCAGATGGGGCCTCGAAGGCAAAGCAGCGAATTCAGCTGGTGGTCTGCGTGGCCGGCCGGTGTAGGGATCTTAACGACTCTTACCGGTTGTGTTCTGCGTAGGTGCCAGTGCACTTTCGTTGTTTGTACGCCACTGCCGTTGAGTGAGCATCCGTCGGGTTGACGTGTGAAGCCTTACTCCTTCACGGGCGTAGGTGCTTACAGTTGACTTTGTACGCGTTTGCACCTCAACCGGTCATGTCAGCATGTGCCAGCGTTTGCGTTGGGCCGGTGCGCTCTGTCGGTTGTCGTGGGATGACGAGCTTGCTCGGCTCCTCGATGATTAGTAGAATCGTGCTGGTTTTCAACGTCGGTACATTGTAGATTCGGTGGCGAGTAGACGGCTGCCCATCTAACCCGTCTTGAAACAC |
|  | >Seq6 [organism=Macrobiotus basiatus] [isolate=USA/NEL/6] 28S ribosomal RNA gene, partial sequence | **MT488402** | TAAGCATATTACTAAGCGGAGGAAAAGAAACCAACGGGGATGCCGATAGTAACTGCGAGTGAAATCGGCCAAGCCCAGCGCCGAATCCTATTGCTGGCAACGGTGACGGGAACTGTGGCGTGAAGAACGCTCTTACCGGTACGGTTCGTGTGCGTAAGTTCTCCTGAGTGAGGCTCCATCCCAGTGAGGGTGCAAGGCCCGTATCGCGCACAACTGGTACCGGTGTAAGTGTTCGGAGAGTCGCCTTGTTTGTGAGTACAAGGTGAAGTCGGTGGTAAACTCCATCGAAGGCTAAATATGACCACGAGTCCGATAGCGAACAAGTACCGTGAGGGAAAATTGAAAAGCACTTTGAAGAGAGAGCGAAACAGTGCGTGAAACCGCTCAGAGGCAAGCAGATGGGGCCTCGAAGGCAAAGCAGCGAATTCAGCTGGTGGTCTGCGTGGCCGGCCGGTGTAGGGATCTTAACGACTCTTACCGGTTGTGTTCTGCGTAGGTGCCAGTGCACTTTCGTTGTTTGTACGCCACTGCCGTTGAGTGAGCATCCGTCGGGTTGACGTGTGAAGCCTTACTCCTTCACGGGCGTAGGTGCTTACAGTTGACTTTGTACGCGTTTGCACCTCAACCGGTCATGTCAGCATGTGCCAGCGTTTGCGTTGGGCCGGTGCGCTCTGTCGGTTGTCGTGGGATGACGAGCTTGCTCGGCTCCTCGATGATTAGTAGAATCGTGCTGGTTTTCAACGTCNGTACATTGTAGATTCNGNGGCGAGTAGACGGCTGCCCATCTAACCCGTCTTGAAACACG |
| ITS2 | >Seq1 [organism=Macrobiotus basiatus] [isolate=USA/NEL/1] internal transcribed spacer 2, partial sequence; 28S ribosomal RNA gene, partial sequence | **MT505165** | TTGCAGGACTTTGTGAACGTTAATTCTTCGAACGCACATTGCGGCTTTGGGTTGACTGAAGCCATGCCTGGTTGAGGGTCAGTTGAAAAACTAGACTCGTAGTCGTAAGCAAAAGACTACGGATTGTCCGCTTAACGAGCCCTGGTGCCGTTTTCGGATCAAGTTGAGACCAGATGTGTGCGCTCATTGCTGGTGATAGCGTTACCAAAACGCTTTGCTAGTTGGAGCATTCGGCTTTCTTGGCCGTGCGCCGCAGCTGCACAAGCGCCAAGACTGCTTACCAACTGCAAGGTGTGGTTTCACCGGTAGGAGCGCAACGCGTAGACGCATCTGTGAAGCAAACACGGCTCGATACGGACAGCTTTGCCGTTTCACGAGACGTATCAATCATTCTTTGACCTCAGCTCAGACAAGATTACCCGCTGAACTTA |
|  | >Seq2 [organism=Macrobiotus basiatus] [isolate=USA/NEL/2] internal transcribed spacer 2, partial sequence; 28S ribosomal RNA gene, partial sequence | **MT505166** | ATTGCAGGACTTTGTGAACGTTAATTCTTCGAACGCACATTGCGGCTTTGGGTTGACTGAAGCCATGCCTGGTTGAGGGTCAGTTGAAAAACTAGACTCGTAGTCGTAAGCAAAAGACTACGGATTGTCCGCTTAACGAGCCCTGGTGCCGTTTTCGGATCAAGTTGAGACCAGATGTGTGCGCTCATTGCTGGTGATAGCGTTACCAAAACGCTTTGCTAGTTGGAGCATTCGGCTTTCTTGGCCGTGCGCCGCAGCTGCACAAGCGCCAAGACTGCTTACCAACTGCAAGGTGTGGTTTCACCGGTAGGAGCGCAACGCGTAGACGCATCTGTGAAGCAAACACGGCTCGATACGGACAGCTTTGCCGTTTCACGAGACGTATCAATCATTCTTTGACCTCAGCTCAGACAAGATTACCCGCTGAACTTA |
|  | >Seq3 [organism=Macrobiotus basiatus] [isolate=USA/NEL/3] internal transcribed spacer 2, partial sequence; 28S ribosomal RNA gene, partial sequence | **MT505167** | TTGCAGGACTTTGTGAACGTTAATTCTTCGAACGCACATTGCGGCTTTGGGTTGACTGAAGCCATGCCTGGTTGAGGGTCAGTTGAAAAACTAGACTCGTAGTCGTAAGCAAAAGACTACGGATTGTCCGCTTAACGAGCCCTGGTGCCGTTTTCGGATCAAGTTGAGACCAGATGTGTGCGCTCATTGCTGGTGATAGCGTTACCAAAACGCTTTGCTAGTTGGAGCATTCGGCTTTCTTGGCCGTGCGCCGCAGCTGCACAAGCGCCAAGACTGCTTACCAACTGCAAGGTGTGGTTTCACCGGTAGGAGCGCAACGCGTAGACGCATCTGTGAAGCAAACACGGCTCGATACGGACAGCTTTGCCGTTTCACGAGACGTATCAATCATTCTTTGACCTCAGCTCAGACAAGATTACCCGCTGAACTTA |
|  | >Seq4 [organism=Macrobiotus basiatus] [isolate=USA/NEL/4] internal transcribed spacer 2, partial sequence; 28S ribosomal RNA gene, partial sequence | **MT505168** | ATTGCAGGACTTTGTGAACGTTAATTCTTCGAACGCACATTGCGGCTTTGGGTTGACTGAAGCCATGCCTGGTTGAGGGTCAGTTGAAAAACTAGACTCGTAGTCGTAAGCAAAAGACTACGGATTGTCCGCTTAACGAGCCCTGGTGCCGTTTTCGGATCAAGTTGAGACCAGATGTGTGCGCTCATTGCTGGTGATAGCGTTACCAAAACGCTTTGCTAGTTGGAGCATTCGGCTTTCTTGGCCGTGCGCCGCAGCTGCACAAGCGCCAAGACTGCTTACCAACTGCAAGGTGTGGTTTCACCGGTAGGAGCGCAACGCGTAGACGCATCTGTGAAGCAAACACGGCTCGATACGGACAGCTTTGCCGTTTCACGAGACGTATCAATCATTCTTTGACCTCAGCTCAGACAAGATTACCCGCTGAACTTA |
|  | >Seq5 [organism=Macrobiotus basiatus] [isolate=USA/NEL/5] internal transcribed spacer 2, partial sequence; 28S ribosomal RNA gene, partial sequence | **MT505169** | GCAGGACTTTGTGAACGTTAATTCTTCGAACGCACATTGCGGCTTTGGGTTGACTGAAGCCATGCCTGGTTGAGGGTCAGTTGAAAAACTAGACTCGTAGTCGTAAGCAAAAGACTACGGATTGTCCGCTTAACGAGCCCTGGTGCCGTTTTCGGATCAAGTTGAGACCAGATGTGTGCGCTCATTGCTGGTGATAGCGTTACCAAAACGCTTTGCTAGTTGGAGCATTCGGCTTTCTTGGCCGTGCGCCGCAGCTGCACAAGCGCCAAGACTGCTTACCAACTGCAAGGTGTGGTTTCACCGGTAGGAGCGCAACGCGTAGACGCATCTGTGAAGCAAACACGGCTCGATACGGACAGCTTTGCCGTTTCACGAGACGTATCAATCATTCTTTGACCTCAGCTCAGACAAGATTACCCGCTGAACTTA |
|  | >Seq6 [organism=Macrobiotus basiatus] [isolate=USA/NEL/6] internal transcribed spacer 2, partial sequence; 28S ribosomal RNA gene, partial sequence | **MT505170** | ATGCAGGACTTTGTGAACGTTAATTCTTCGAACGCACATTGCGGCTTTGGGTTGACTGAAGCCATGCCTGGTTGAGGGTCAGTTGAAAAACTAGACTCGTAGTCGTAAGCAAAAGACTACGGATTGTCCGCTTAACGAGCCCTGGTGCCGTTTTCGGATCAAGTTGAGACCAGATGTGTGCGCTCATTGCTGGTGATAGCGTTACCAAAACGCTTTGCTAGTTGGAGCATTCGGCTTTCTTGGCCGTGCGCCGCAGCTGCACAAGCGCCAAGACTGCTTACCAACTGCAAGGTGTGGTTTCACCGGTAGGAGCGCAACGCGTAGACGCATCTGTGAAGCAAACACGGCTCGATACGGACAGCTTTGCCGTTTCACGAGACGTATCAATCATTCTTTGACCTCAGCTCAGACAAGATTACCCGCTGAACT |
| 18S rRNA | >Seq1 [organism=Macrobiotus basiatus] [isolate=USA/NEL/1] 18S ribosomal RNA gene, partial sequence | **MT498094** | GTCTCAAAGATTAAGCCATGCATGTCTCAGTACTTGCTTTTACAAGGCGAAACCGCGAATGGCTCATTAAATCAGTTATGGTTCACTGGATCGTTAATTTTACACGGATAACTGTGGTAATTCTAGAGCTAATACGTGCAAATAGCTCGCTTCCTTGTGGAGTGAGCGCAGTTATTAGATCAAAACCAATCCGGCCTTCGGGTCGGTACATTTGGTGACTCTGAATAACCGAAGCGGAGCGCATGGTCTCGTACCGGCGCCAGATCTTTCAAGTGTCTGACTTATCAGCTTGTTGTTAGGTTACGTTCCTAACAAGGCTTCAACGGGTAACGGGGTATCAGGGTCCGATACCGGAGAGGGAGCCTGAGAAACGGCTACCACATCCAAGGAAGGCAGCAGGCGCGCAAATTACCCACTCCTAGCACAGGGAGGTAGTGACGAAAAATAACGATGCGAGGGCTATTAGCTTCTCGTAATCGGAATGGGTACACTTTAAATCCTTTAACGAGGATCTATTGGAGGGCAAGTCTGGTGCCAGCAGCCGCGGTAATTCCAGCTCCAATAGCGTATATTAAAGTTGCTGCGGTTAAAAAGCTCGTAGTTGGATCTGGGCTTCTGAATGGATGGTGCACTTTTCGGTGCAACTGTTGGTTTGGTGCCACATGCCGGCCATGTCTTGCATGCTCTTCATTGAGTGTGCATGGCGACCGGAACGTTTACTTTGAAAAAATTAGAGTGCTCAAAGCAGGCGTATGGCCTTGCATAATGGTGCATGGAATAATGGAATAGGACCTCGGTTCTATTTTGTTGGTTTTCGGAACTCGAGGTAATGATTAAGAGGAACAGACGGGGGCATTCGTATTGCGGCGTTAGAGGTGAAATTCTTGGATCGTCGCAAGACGAACTACTGCGAAAGCATTTGCCAAGAATGTTTTCATTAATCAAGAACGAAAGTTAGAGGTTCGAAGGCGATCAGATACCGCCCTAGTTCTAACCATAAACGATGCCAACCAGCGATCCGTCGGTGTTCTTTTGTTGACTCGACGGGCAGCTTTCCGGGAAACCAAAGTGTTTAGGTTCCGGGGGAAGTATGGTTGCAAAGCTGAAACTTAAAGGAATTGACGGAAGGGCACCACCAGGCGTGGAGCCTGCGGCTTAATTTGACTCAACACGGGAAAACTTACCCGGCCCGGACACTGTAAGGATTGACAGATTGAGAGCTCTTTCTTGATTCGGTGGGTGGTGGTGCATGGCCGTTCTTAGTTGGTGGAGCGATTTGTCTGGTTAATTCCGATAACGAACGAGACTCTAGCCTGCTAAATAGCCAACCGATCCGCAGCGTCGGTTGCTACAAAAGCTTCTTAGAGGGACAGGCGGCGTTTAGTCGCACGAGATTGAGCAATAACAGGTCTGTGATGCCCTTAGATGTCCGGGGCCGCACGCGCGCTACACTGAAGGGACCAGAGTGCTTAATCACCTTGGCCGGAAGGCCTGGGGAATCCGATTAAACCCCTTCGTGATTGGGATTGAGCTTTGTAATTATCGCTCATGAACGAGGAATGCCCAGTACTCGCGAGTCATAAGCTCGCGATGATTACGTCCCTGCCCTTTGTACACACCGCCCGTCGCTACTACCGATTGAATGATTTAGTGAGGTCTTCGGACTGGCCATCGAGGCTGACTCTGTTGGCTTCGGTTGGATCGGAAAGACGACCAAAC |
| COI | >Seq1 [organism=Macrobiotus basiatus] [isolate=USA/NEL/1] cytochrome oxidase subunit I (COI) gene, partial cds; mitochondrial | **MT502116** | CAAGACATGCATTTATTATAATTTTTTTTTTTGTAATACCTATTCTAATTGGAGGGTTCGGCAATTGACTCGTTCCCTTAATAATTGGAGCTCCTGATATAGCTTTCCCTCGAATAAATAATTTAAGGTTTTGACTTCTCCCCCCATCGTTTACCCTTATTATAACAAGTACTATAAGAGAACAAGGAGCAGGTACAGGATGAACTGTATACCCCCCCCTATCCCATTTTTTTGCCCATAGAGGACCGAGAGTTGATTTAACAATCTTTTCTCTTCATATCGCAGGAGTATCGTCAATTCTAGGAGCTATTAATTTTATTTCTACTATTATTAATATACGAGCTCCAAACCTATCTCTAGAAAATATGCCTTTATTTGTATGATCTGTCCTAATTACCGCAATCCTTTTACTTTTAGCTTTACCTGTATTGGCAGGAGGAATCACAATACTACTAATAGACCGTAATTTCAACACTTCTTTTTTTGATCCTGCAGGAGGAGGAGACCCTATTTTATACCAACATTTATTC |
|  | >Seq2 [organism=Milnesium inceptum] [isolate=USA/Nelson/1] cytochrome oxidase subunit I (COI) gene, partial cds; mitochondrial | **MT502117** | AAAGATATTGGTATATTGTATTTTATTTTTGGTATTTGATGTGCTTTTGTAGGTTCAGGTTTAAGTGTGTTAATTCGTCTTGAATTATCTCAGCCTAACACAATATTAATAAGTGAAGATATTTATAATGCTTTTATTACAAGTCATGCTTTAGTAATGATTTTTTTTTTTGTTATACCTGTTTTAATTGGAGGTTTTGGAAATTGATTAGTTCCTCTTATAATTAGATCACCAGATATAGCTTTTCCTCGTATTAATAATGTAAGATTTTGAATATTAGTTGCTTCTTTTGGTTTGTTGCTTTTTAGAATATTTAGGGGTACAGGAGTAGGAGCTGGTTGAACACTATATCCTCCGTTAACTAGGTATAATGGCCATAGCAGTCATGCTGTCGATTATGCAATTTTGTCTTTACATATTGCAGGAGCATCGTCAATTTTTAGTGCACTGAATTTTTTAACGACGATTATTAATATACACTATTTTGGAGTACGAATAGATAAATTACCGTTGTTTGTGTGATCGATTTTTATTACTGCTCTATTGTTAGTTTTGGCTTTACCAGTACTTGCTGGAGCAATTACAATATTAATTTCTGATCGTAATTTCACTACTACATTTTTTGATCCGGCAGGGGGAGGAGATCCTGTTTTATTTCAACATTTATTTTGNTTTTTTGG |
